# An NMR and MD study of complexes of bacteriophage lambda lysozyme with tetra‐ and hexa‐N‐acetylchitohexaose

**DOI:** 10.1002/prot.25770

**Published:** 2019-07-26

**Authors:** Aysegul Turupcu, Alice M. Bowen, Alexandre Di Paolo, André Matagne, Chris Oostenbrink, Christina Redfield, Lorna J. Smith

**Affiliations:** ^1^ Institute of Molecular Modeling and Simulation, University of Natural Resources and Life Sciences Vienna Vienna Austria; ^2^ Department of Chemistry University of Oxford Oxford UK; ^3^ Laboratoire d'Enzymologie et Repliement des Protéines, Centre d'Ingénierie des Protéines Institut de Chimie, Université de Liège Liège Belgium; ^4^ Department of Biochemistry University of Oxford Oxford UK

**Keywords:** ligand binding, lysozymes, molecular dynamics, NMR spectroscopy, oligosaccharides

## Abstract

The X‐ray structure of lysozyme from bacteriophage lambda (λ lysozyme) in complex with the inhibitor hexa‐N‐acetylchitohexaose (NAG6) (PDB: 3D3D) has been reported previously showing sugar units from two molecules of NAG6 bound in the active site. One NAG6 is bound with four sugar units in the ABCD sites and the other with two sugar units in the E′F′ sites potentially representing the cleavage reaction products; each NAG6 cross links two neighboring λ lysozyme molecules. Here we use NMR and MD simulations to study the interaction of λ lysozyme with the inhibitors NAG4 and NAG6 in solution. This allows us to study the interactions within the complex prior to cleavage of the polysaccharide. ^1^H^N^ and ^15^N chemical shifts of λ lysozyme resonances were followed during NAG4/NAG6 titrations. The chemical shift changes were similar in the two titrations, consistent with sugars binding to the cleft between the upper and lower domains; the NMR data show no evidence for simultaneous binding of a NAG6 to two λ lysozyme molecules. Six 150 ns MD simulations of λ lysozyme in complex with NAG4 or NAG6 were performed starting from different conformations. The simulations with both NAG4 and NAG6 show stable binding of sugars across the D/E active site providing low energy models for the enzyme‐inhibitor complexes. The MD simulations identify different binding subsites for the 5th and 6th sugars consistent with the NMR data. The structural information gained from the NMR experiments and MD simulations have been used to model the enzyme‐peptidoglycan complex.

**Abbreviations**
BMRB: Biological Magnetic Resonance Data Bank
HSQC: heteronuclear single quantum correlation
MD: molecular dynamics
NAG: N‐acetylglucosamine
NAG4: tetra‐N‐acetylchitohexaose
NAG6: hexa‐N‐acetylchitohexaose
NAM: N‐acetylmuramic acid
NMR: nuclear magnetic resonance
PDB: Protein Data Bank
SPC: simple point charge
T4L: lysozyme from T4 bacteriophage

## 1 INTRODUCTION

Lysozymes are very widespread throughout nature, being found in phage, bacteria, plants, and animals.^1^ Along with the lytic transglycosylases they are collectively known as N‐acetyl‐β‐D‐muramidases cleaving the β‐1,4‐glycosidic bond between N‐acetylmuraminic acid (NAM) and N‐acetylglucosamine (NAG) of the bacterial peptidoglycan.^2^ The difference between these enzymes is in the cleavage mechanism. Lytic transglycosylases form a 1,6‐anhydromuramic acid product by an intramolecular transglycosylation reaction while lysozymes in general perform a hydrolysis reaction.^3^ However, lysozyme from bacteriophage lambda (λ lysozyme) cleaves the peptidoglycan without the intervention of a water molecule. This mechanism therefore differs from that observed with most of the lysozymes and makes λ lysozyme a lytic transglycosylase.^4^

**Figure 1:**
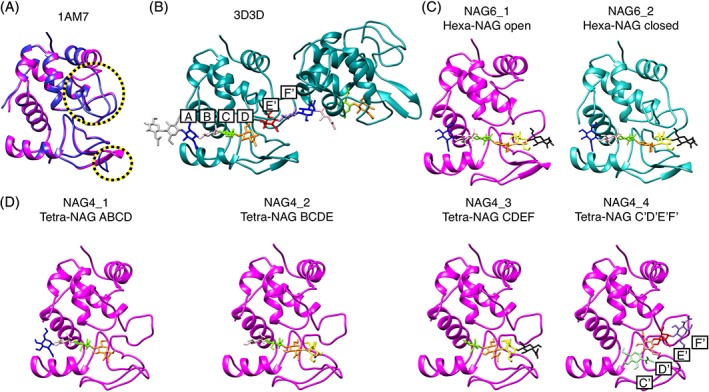
The structure of λ lysozyme in the available X‐ray structures and the modeled initial structures used in the MD simulations. A, The X‐ray structure of the apo state of λ lysozyme (1AM7). The three molecules in the asymmetric unit are shown superimposed. One of these is in the closed conformation (blue) and the other two are in the open conformation (magenta). The upper lip (residues 128‐141) and lower lip (51‐60) regions of λ lysozyme (indicated by yellow/black circles) show significant differences between the open and closed conformations. B, The crystal structure containing two molecules of the complex of λ lysozyme in the closed conformation with two NAG6 molecules (PDB ID: 3D3D). Each molecule is bound to its NAG6 unit using the A, B, C, and D subsites and to the other sugar unit using its E′ and F′ subsites. C, The modeled open (magenta) and closed (cyan) NAG6‐lysozyme systems used as the initial structures in the NAG6_1 and NAG6_2 simulations, respectively. Here, the 5th and 6th sugars occupy different subsites on the protein than in panel B. The model was created by keeping the first four sugars in sites ABCD and adding the 5th and 6th sugars to sites E and F with their glycosidic dihedral angles set to their lowest energy state (see Section 2). D, The four different initial NAG4‐lysozyme models used in the simulations with sugars occupying sites ABCD (NAG4_1), BCDE (NAG4_2), CDEF (NAG4_3), and C′D′E′F′ (NAG4_4). All four simulations started from the open structure of λ‐lysozyme (shown in magenta). In all panels, the sugar units are color‐coded as follows: A‐blue, B‐pale pink, C‐green, C′‐pale green, D‐orange, D′‐pale orange, E‐yellow, E′‐red, F‐black, F′‐lavender. Sugar units not bound to λ lysozyme are shown in pale gray in B)

The active site of λ lysozyme is located between an upper and a lower domain (Figure 1). The upper domain contains four α helices (α1, α4, α5, and α6) and a long loop region that forms the upper lip. The lower domain is made up of a helix (α2) and a β‐sheet region (β1‐6) with loops. The two domains are connected by helix α3. The crystal structure of the apo form of the enzyme (PDB ID:1AM7^5^) contains three molecules in the unit cell; one of them (chain B) is in a closed conformation and the other two (chains A and C) are open. The main differences between these conformations are the positions of the upper (residues 128‐141) and the lower (residues 51‐60) lip regions (Figure 1A). In the open conformation of the enzyme, the active‐site cleft is wide open, while in the closed conformation the upper lip moves toward the lower domain restricting access to the active‐site cleft. The α6 helix partly unwinds in the closed conformation; this makes the upper lip region longer which enables it to further cover the active‐site region. NMR studies and MD simulations^6^, ^7^ of the apo form of λ lysozyme have shown that in solution, rather than adopting either the open or closed structure, λ lysozyme populates an ensemble of conformations resulting from the dynamic nature of the upper and lower lip regions. The NMR data for apo λ lysozyme show that in solution it populates the open conformation more than the closed conformation.

A crystal structure of λ lysozyme in complex with hexa‐N‐acetylchitohexaose (NAG6), an inhibitor, has been determined (PDB ID: 3D3D,^8^ Figure 1B). The crystallographic unit cell of this structure contains two molecules of λ lysozyme (chains A and B) (Figure S1). Both molecules have a similar structure to the closed molecule of the apo form of the enzyme. The active site of lysozyme is traditionally divided into six subsites, A‐F, where each subsite can be occupied by one sugar unit. In the crystal structure of the complex of λ lysozyme with NAG6 there are two NAG6 molecules within the unit cell. The NAG6 unit of molecule A binds to both protein molecules; it occupies the first four subsites (A‐D) of protein molecule A and the last two subsites (E′ and F′) of the noncrystallographically related neighboring protein molecule B (Figure 1B). This structure, which lacks a glycosidic bond across the D‐E′ subsites, potentially represents the products of the cleavage reaction.

In this work we have used experimental NMR studies and MD simulations to study the interaction of NAG4 and NAG6 with λ lysozyme in solution. It has been shown previously that NAG4 and larger NAG polymers are able to act as competitive inhibitors of the cleavage reaction of the natural substrate peptidoglycan.^8^ Our work enables us to investigate different possible sugar binding subsites within λ lysozyme that could be occupied prior to and during catalysis and the effects of sugar binding on the dynamics of the protein. Using the insights gained from the NMR and MD studies, the complex of λ lysozyme with the natural peptidoglycan substrate has been modeled.

## 2 MATERIALS AND METHODS

### 2.1 NMR titrations

^15^N‐labeled λ lysozyme was expressed and purified as described previously.^9^ NMR experiments were performed at a λ lysozyme concentration of 0.24 mM in a 95% H_2_O/5% D_2_O sodium phosphate buffer at pH 4.6 and 20°C. HSQC spectra were collected using home‐built spectrometers controlled with GE/Omega software and equipped with Oxford Instruments Company magnets and home‐built triple‐resonance pulsed‐field‐gradient probe heads. Titrations with NAG4 and NAG6 were carried out at ^1^H operating frequencies of 500.10 and 750.04 MHz, respectively. The data sets were acquired using 128 complex t_1_ increments with ^15^N sweep widths of 1515.15 and 2272.73 Hz at ^15^N frequencies of 50.68 and 76.01 MHz, respectively. 1 K complex data points were recorded in the F_2_ dimension with sweep widths of 7142.86 and 10526.32 Hz at 500 and 750 MHz, respectively. Eighty and 96 scans were collected per t_1_ increment for the HSQC experiments at 500 and 750 MHz, respectively. The NMR spectra in the presence of NAG4 and NAG6 were assigned and analyzed using CcpNmr Analysis^10^ using resonance assignments for λ lysozyme published previously (BMRB 16664).^9^

The interaction of λ lysozyme with NAG4 and NAG6 was monitored using 2D ^1^H‐^15^N HSQC spectra. NAG4 and NAG6, derived by the hydrolysis of crab shell chitin, were purchased from Seikagaku Corporation. The first titration was carried out using NAG6, the inhibitor used in the X‐ray crystallography studies.^8^ Titration data for NAG6 were collected at concentrations of up to 6 mM in 1 mM increments. Concentrations above ~6 mM were not possible due to the limited solubility of the NAG6. In order to achieve higher inhibitor concentrations and to investigate the binding mode of a shorter inhibitor, a second set of titration experiments was collected using NAG4. For the NAG4 titrations, lower initial concentrations of NAG4 were used, in the light of the significant broadening of peaks in the 1 mM NAG6 spectrum, and higher NAG4 concentrations were achievable due to the higher solubility of NAG4 (concentrations were 0, 0.2, 0.4, 0.6, 0.8, 1.2, 2.0, 3.0, 4.2, 6.2, 9.0, 12.4, 16.2, and 26.0 mM).

### 2.2 Chemical shift perturbation and dissociation constant calculation

The combined ^1^H^N^ and ^15^N chemical shift difference (*δ*) was calculated for each residue using the standard equation ($\delta =\sqrt{\frac{1}{2}\left[\delta_{H}^{2}+{\left(\alpha \delta_{N}\right)}^{2}\right]\;}$); the ^15^N shift changes were scaled by a factor of *α* = 0.15 compared to the ^1^H^N^ chemical shift changes.^11^ For the calculation of the dissociation constant the combined chemical shift difference is described as a function of the total sugar concentration (*L*_T_) by

(1) $${\Delta \delta }_{\mathrm{obs}}=\frac{{\Delta \delta }_{\max}}{2P_{T}}\left(K_{d}+L_{T}+P_{T}\right)-\sqrt{{\left(K_{d}+L_{T}+P_{T}\right)}^{2}-4L_{T}P_{T}}$$

where *P*_T_ is the total protein concentration, *K*_d_ is the dissociation constant and Δ*δ*_max_ is the maximum observed chemical shift difference. In the *K*_d_ calculations, the average of the combined chemical shift changes from the residues showing evidence of hydrogen bonding interactions detected from both the simulations and the NMR experiments were used. Neighboring residues that showed significant changes through inductive effects were also included. When all the subsites were considered, the residues used in the K_d_ calculations were 19, 20, 68, 69, 73, 77, 98, 101, and 102. To decide which combined chemical shift changes are large enough to be considered significant indicators of the binding site, the method from Schumann et al^12^ was used.

### 2.3 Molecular dynamics (MD) simulations

MD simulations of λ lysozyme were performed using the GROMOS11 biomolecular simulation package (http://www.gromos.net)^13^ and the 54A8 GROMOS force field.^14^ The carbohydrate content of the complex was parameterized with the 53A6glyc parameter set^15^ of the GROMOS force field for carbohydrates. Parameters for the N‐acetyl group in NAG have been adjusted to the current protein force field previously.^16^ For the modeling of the initial conformations, two crystal structures of λ lysozyme with PDB ID: 3D3D^8^ (molecule B) and 1AM7^5^ (molecule A) were used as closed and open conformations, respectively. The initial structure of the hexasaccharide bound to the closed λ lysozyme was modeled by using the crystallographic NAG6 conformation for sugars 1 to 4 which occupy subsites A to D. The tetrasaccharide was then extended by adding two NAG units with PyMOL.^17^ While adding these two NAG residues, the glycosidic angles at the linkage were set according to their most favorable energetic state with *φ*, *ψ* angles of −84°, 102° which have been identified previously.^16^ This places the final two sugar units 5 and 6 into subsites that we refer to as E and F while the subsites populated by the second NAG6 molecule in the crystal structure are referred to as E′ and F′ (Figure 1B). It is not possible to place the final two sugar units in the E′ and F′ sites while retaining a low‐energy covalent bond between sugar 4, in site D, and sugar 5, in site E′. For the modeling of the open structure, molecule A from the crystal structure with PDB ID: 1AM7^5^ was aligned on the structure of closed λ lysozyme with NAG6 bound.

In addition to the complexes of open and closed λ lysozyme with NAG6, complexes of λ lysozyme with NAG4 were modeled. Due to the availability of the six subsites, four simulations of the λ lysozyme‐NAG4 complex system were run, all starting from an open conformation of λ lysozyme. In the different initial structures used for these simulations the sugars occupy subsites ABCD, BCDE, CDEF, and C′D′E′F′. Here, E′ and F′ subsites refer to the subsites which were observed in the crystal structure, as described above. When the last two sugars were fitted to the E′ and F′ subsites, the first two sugars needed to be shifted from subsites C and D to subsites we refer to as C′ and D′ in order to adopt glycosidic angles with favorable energies.

During the structure determination, all the Trp residues in the 1AM7 crystal structure were replaced by aza‐tryptophans. These were changed to Trp residues for the simulations. Hydrogen atoms were added according to geometric criteria followed by a short energy minimization in vacuo using the steepest‐descent algorithm. Each protein complex with its oligosaccharides was solvated with SPC water^18^ in a periodic cubic box with a minimum protein‐to‐wall distance of 1.4 nm. Eight Cl^−^ ions were added to make the system charge neutral. Systems were further relaxed by a steepest descent minimization with position restraints on the solute atoms. For the equilibration, initial random velocities of all atoms were assigned from a Maxwell‐Boltzmann distribution at 60 K, and the system was heated up to 300 K by increasing the temperature of the external bath by 60 K every 20 ps while simultaneously, position restraints on the solute atoms were reduced from 2.5 × 10^4^ to 0.0 kJ mol^−1^ nm^−2^. All the simulations were performed at a constant temperature of 300 K and a constant pressure of 1 atm using a weak coupling scheme^19^ for both temperature and pressure with coupling times *τ*_T_ = 0.1 ps and *τ*_P_ = 0.5 ps and an isothermal compressibility of 4.575 × 10^−4^ kJ^−1^ mol nm^3^. For all production runs, a leapfrog integration scheme^20^ with a time step of 2 fs was used, and covalent bonds were constrained to maintain the optimal bond length. Nonbonded interactions were computed using a pairlist^21^ that was updated every 5 steps. Interactions up to 0.8 nm were computed at every time step and up to 1.4 nm were computed at pair list updates and kept constant in between. Long‐range electrostatic interactions beyond a cutoff of 1.4 nm were truncated and approximated by a generalized reaction field^22^ with a dielectric permittivity of 61.^23^ The SHAKE algorithm^24^ was used to maintain the bond lengths.

For the clustering and time series analysis the GROMOS++ software^25^ was used. The DSSP program^26^ was used to identify the regions of secondary structures of the conformers within the simulations. For the hydrogen bond analysis, a geometrical criterion was used where hydrogen bonds are identified if the hydrogen‐acceptor distance is smaller than 0.25 nm and the donor‐hydrogen‐acceptor angle is larger than 135°. Conformational clustering was performed based on pairwise root‐mean‐square differences between configurations, using a cutoff of 0.35 nm to identify structural neighbors.^27^

## 3 RESULTS AND DISCUSSION

### 3.1 NMR chemical shift perturbations

**Figure 2:**
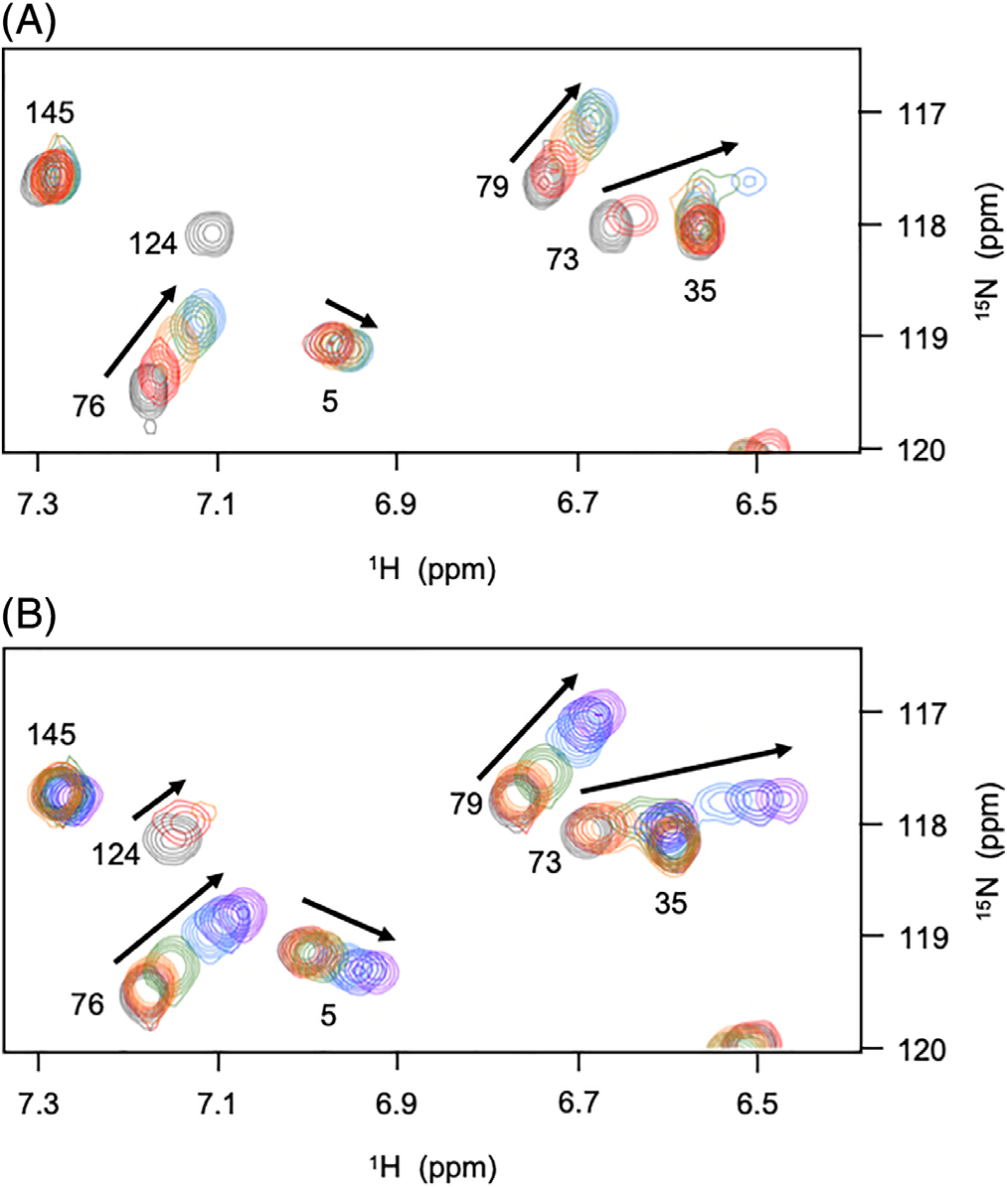
Superposition of a region of the ^1^H‐^15^N HSQC spectra of λ lysozyme recorded during titrations with (A) NAG6 and (B) NAG4. Peaks arising from Asn5, Val35, Trp73, Ala76, Lys79, Trp124, and Lys145 are shown. The arrows indicate the direction of the peak shifts during the course of the titrations. A, Overlaid spectra correspond to NAG6 concentrations of 0 (dark gray), 1 mM (red), 2 mM (orange), 4 mM (green), and 6 mM (blue). B, Overlaid spectra correspond to NAG4 concentrations of 0 (dark gray), 0.4 mM (red), 0.8 mM (orange), 2 mM (green), 6 mM (blue), 12.4 mM (dark blue), and 26 mM (purple). It can be seen that peaks shift in the same direction and to a very similar extent in the 6 mM spectra for NAG6 and NAG4 shown in blue. The peak corresponding to Trp124 which disappears with 1 mM NAG6 can be seen in the spectra collected with 0.4 and 0.8 mM NAG4 [Color figure can be viewed at http://wileyonlinelibrary.com]

**Figure 3:**
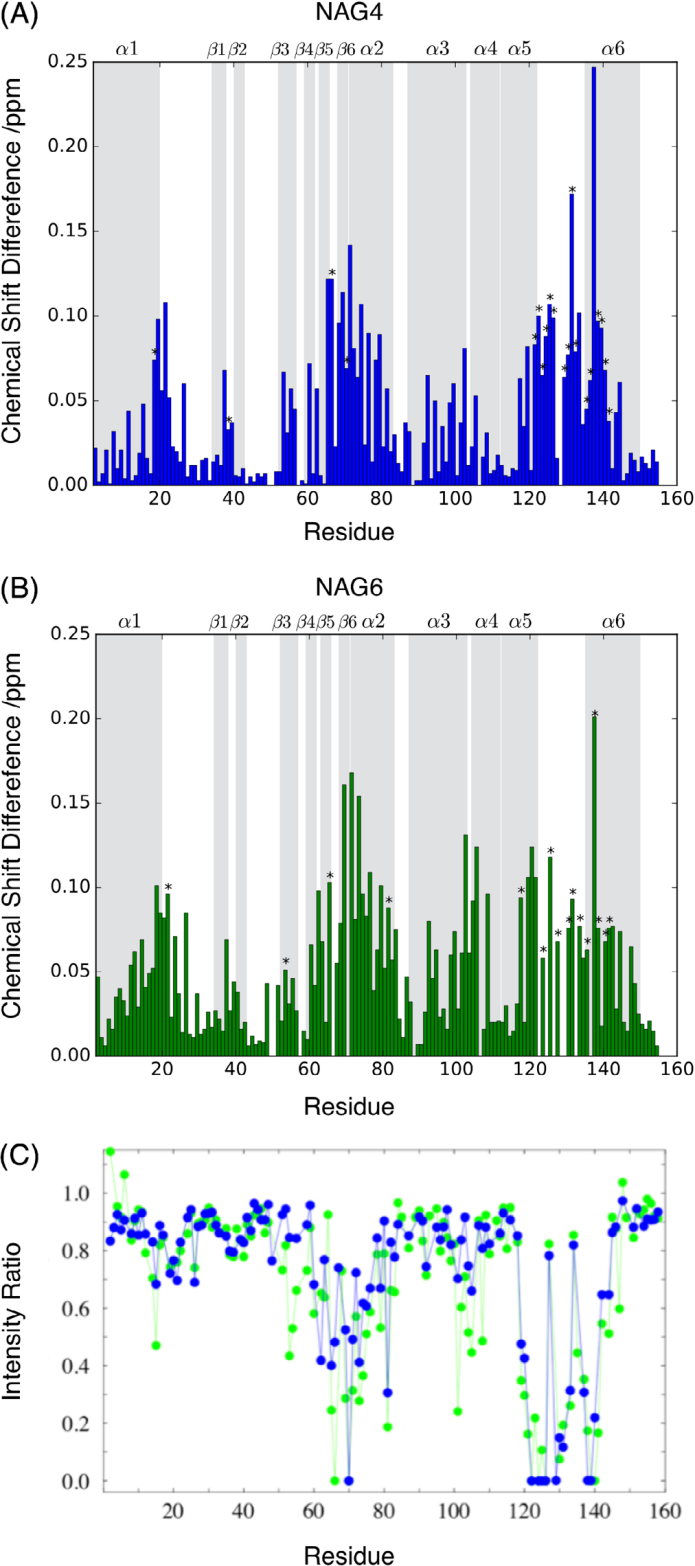
Comparison of chemical shift differences and peak broadening observed with NAG4 and NAG6. Combined ^1^H^N^‐^15^N chemical shift difference between apo λ lysozyme and λ lysozyme in complex with ~6 mM NAG4 (A) and ~6 mM NAG6 (B). The chemical shift differences of peaks that disappear from spectra have been extrapolated, where possible, using the experimental *K*_d_ value and are indicated with an asterisk. Regions of secondary structure in λ lysozyme are shown in gray and labeled at the top of each plot. C, HSQC peak intensity for λ lysozyme observed with ~1 mM NAG4 (blue) or NAG6 (green) relative to intensity observed for apo λ lysozyme. Peaks that broaden beyond detection have an intensity ratio of 0 [Color figure can be viewed at http://wileyonlinelibrary.com]

The binding of tetra‐NAG (NAG4) and hexa‐NAG (NAG6) to λ lysozyme in solution was characterized, at a residue‐specific level, using NMR methods. 2D ^1^H‐^15^N HSQC spectra were recorded and analyzed for λ lysozyme in the presence of different concentrations of NAG6 (0‐6 mM) and NAG4 (0‐26 mM); a region of the HSQC spectra is shown in Figure 2. The changes in backbone and side chain ^1^H^N^ and ^15^N chemical shifts and peak intensities were followed through the titrations (Figure 3). Residues without significant chemical shift changes in the presence of 6 mM NAG (such as Val35 and Lys 145 in Figure 2) also showed no significant change in peak shape in the presence of NAG6. This suggests there is no increase in the rotational correlation time indicating that, in solution, the sugar molecules do not cross‐link two λ lysozyme molecules as is seen in the crystal structure of the λ lysozyme‐NAG6 complex.^8^

The spectrum of λ lysozyme in the presence of NAG6 shows peak broadening of specific residues in addition to changes in chemical shifts (Figures 2 and 3); approximately 10 residues are broadened beyond detection with the first addition of ~1 mM NAG6 (Figure 3C) and these cannot be followed during the titration. In the NAG4 titration, smaller additions of the sugar were made allowing more residues to be followed during the course of the titration. A comparison of the loss in peak intensity in the presence of ~1 mM NAG4 and NAG6 shows similar patterns of broadening across the λ lysozyme sequence for the two sugars. A comparison of the spectra shown in Figure 2 for the NAG4 and NAG6 titrations also shows that peaks shift in the same way in the two series of spectra. The combined backbone ^1^H^N^ and ^15^N chemical shift changes for λ lysozyme in the presence of ~ 6 mM NAG4 and NAG6 are shown as a function of sequence in Figure 3. For the majority of residues, shifts of similar magnitude were observed in the two titrations (Figure 3) suggesting that the two sugars bind to λ lysozyme in a similar manner. The broadening and shifts observed for residues including Glu101, Asn122, Leu70, Ala125, Glu19, and Gly20 suggest interactions of NAG4/NAG6 in the A, B, C, D, E, and F sites, respectively. Chemical shift changes observed for NAG4 concentrations ranging from 0.2 to 26 mM allow the dissociation constant to be determined using standard methods. This was calculated to be ~6 mM (Figure S2). The titrations with NAG6 show a similar pattern of chemical shift changes suggesting a similar *K*_d_ value.

### 3.2 Analysis of peak broadening

The most significant broadening of peaks is observed for residues at the C‐terminus of λ lysozyme (Figure 3C). Addition of 1 mM NAG6 leads to broadening beyond detection of the peaks corresponding to residues Asn122, Trp124, Ser126, Ala130, Ala139, and Asp140. The crystal structure of the NAG6‐λ lysozyme complex shows that Asn122, Ile123, Ala125, and Phe135 are involved in direct hydrogen bond interactions with sugars in the B, C, D, and E sites, respectively. In addition, Trp124, Ser126, Tyr132, and Gln134 are within 0.4 nm of sugars in the C, D, and E sites. Therefore, some of the observed broadening is likely to arise from these direct interactions. Interestingly, residues 139 and 140, which are completely broadened, and neighboring residues 138 and 141, which are significantly broadened, do not make contacts with the sugar moieties. However, these residues are significantly perturbed both by large displacements in the structure and by changes in hydrogen bonding interactions between the open and closed structures of the protein.^5^ For example, the H^N^ of Lys138, Ala139, and Asp140 are hydrogen bonded to the CO of Gln134, Phe135, and Glu136, respectively, in the open structure but not in the closed one. Thus, some of the observed peak intensity perturbations for C‐terminal residues may indicate a change in the population of open and closed structures, or in the dynamics of their interconversion, as NAG4/NAG6 bind to λ lysozyme.

### 3.3 Molecular dynamics simulations

**Figure 4:**
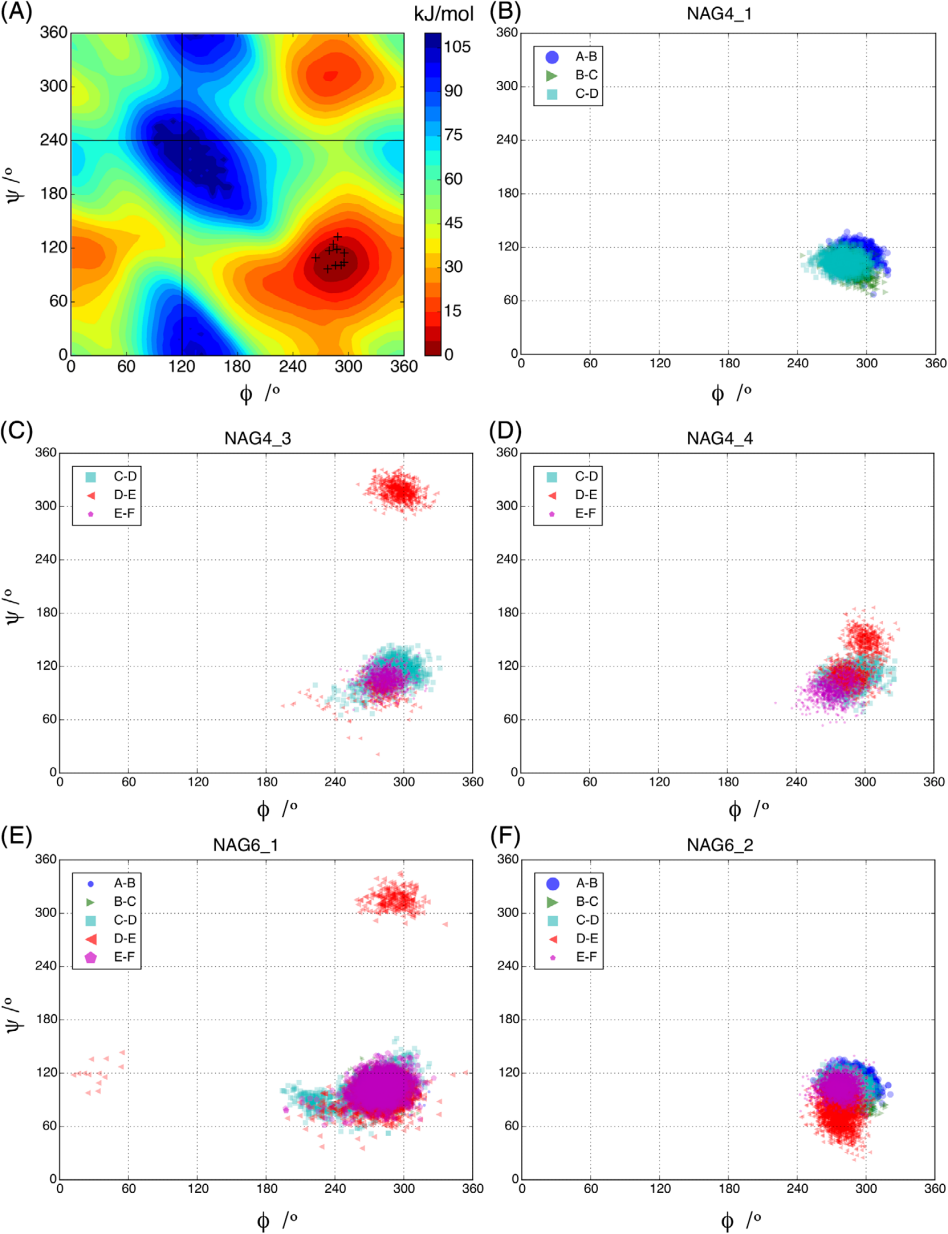
Representation of the glycosidic dihedral angles (*φ*, *ψ*) of the NAG sugar units populating sites A to F. Panel A shows the free‐energy landscape of the β‐GlcNAc‐(1‐4)‐β‐GlcNAc linkage where the red region (0 kJ/mol) shows the lowest free‐energy state. Scatter points indicate the glycosidic dihedral angles of the λ lysozyme‐NAG6 crystal structure (PDB ID:3D3D). They all occupy the same region which is the lowest free‐energy state for this linkage. The glycosidic dihedral angles of the sugar units (populating sites A to F) observed during the MD simulations are plotted for the NAG4_1 (panel B), NAG4_3 (panel C), NAG4_4 (panel D), NAG6_1 (panel E), and NAG6_2 (panel F) simulations. Only the linkage between the sugars populating the D and E sites (shown in red) adopts a conformation corresponding to the second low free energy state [Color figure can be viewed at http://wileyonlinelibrary.com]

MD simulations of λ lysozyme in complex with NAG4 and NAG6 were used to provide structural models for the complex and to characterize the dynamics of the protein complex. Different conformations of λ lysozyme seen in crystallographic studies were used as starting structures for the simulations to capture a dynamic picture of the enzyme. Two 150 ns simulations of λ lysozyme in complex with NAG6 were performed starting from the open and closed conformations of λ lysozyme (Figure 1C; simulations NAG6_1 and NAG6_2, respectively). As discussed above, in the crystal structure subsites ABCD in λ lysozyme are occupied by one NAG6 molecule while the final two subsites are occupied by a second NAG6 molecule from a noncrystallographically related neighboring molecule. For the simulations, the initial structure of the hexasaccharide was modeled by using the crystallographic NAG6 conformation for sugars 1 to 4 which occupy subsites A to D and then adding the two final sugar units with a linkage conformation corresponding to the lowest free energy. This places the final two sugar units 5 to 6 into subsites that we refer to as E and F while the subsites populated by the second NAG6 molecule in the crystal structure are referred to as E′ and F′ (Figure 1B). It is not possible to place the final two sugar units in the E′ and F′ sites while retaining a low‐energy covalent bond between sugar 4, in site D, and sugar 5, in site E′. The free‐energy landscape of the glycosidic linkage is illustrated in Figure 4A^9^ with the glycosidic dihedral angles of the NAG6 units in the bound crystal structure shown in black, populating the lowest energy state. During simulation NAG6_1, a change in the orientation of the linkage between the sugar units populating the D and E subsites was observed (Figure 4E). This change in the glycosidic dihedral angle allows the movement of the sugars 5 and 6 toward the upper domain, populating new positions which we refer to as sites E^u^ and F^u^. This change is not observed when the protein adopts the closed conformation and so different interactions with the protein are seen for sugars 5 and 6 in the simulations starting from the open and closed states of λ lysozyme.

Four 150 ns simulations of λ lysozyme in complex with NAG4 starting from the open conformation of λ lysozyme were performed. In these four simulations the sugar occupied binding subsites ABCD, BCDE, CDEF, and C′D′E′F′ in the starting structure (Figure 1D; simulations NAG4_1 to 4, respectively) where EF and E′F′ are the subsites populated when the lowest free‐energy glycosidic linkage is adopted and those populated in the crystal structure, respectively. When the last two sugars were fitted into the E′F′ subsites it was not possible to place the first two sugars in the C and D subsites. Therefore, the C′D′E′F′ model was created by populating the E′F′ subsites and setting the D′‐E′ glycosidic angle to its lowest energy state (NAG4_4). The C′D′ subsites were shifted compared to those in the crystal structure as can be seen in Figure 1D. A similar binding mode is seen in the complex of Ra‐ChiC, a lysozyme‐like chitinolytic enzyme, with NAG4 (PDB ID 3W6C^28^; Figure S3) which has the highest sequence similarity among the structural representatives of λ lysozyme 29, 30, 31 in the Protein Data Bank (PDB, http://www.rcsb.org 32).

**Figure 5:**
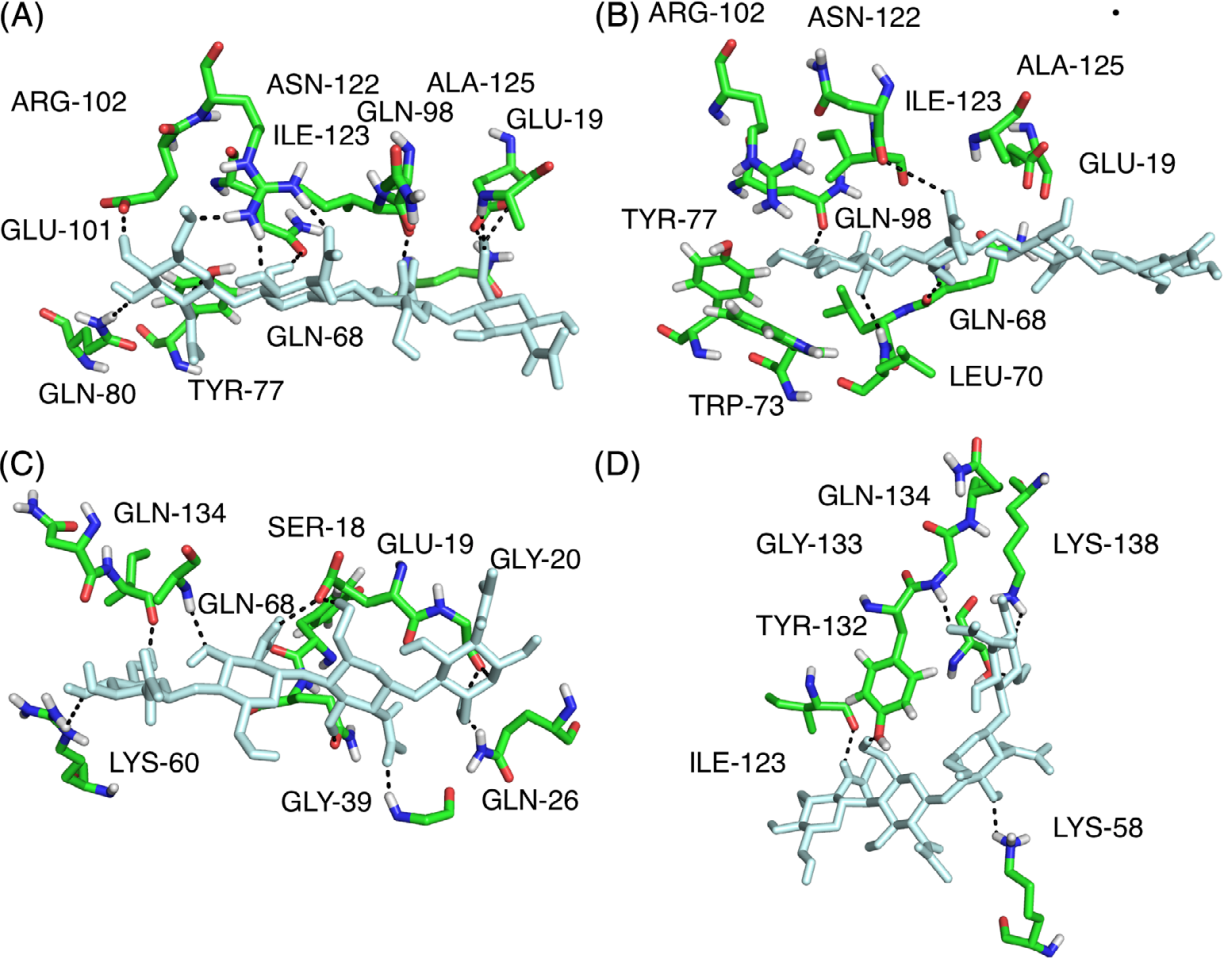
Dynamic hydrogen bonding pattern captured in the MD simulations. Panels show conformations in the λ lysozyme‐NAG4 simulations with the key hydrogen bond interactions between sugar (pale cyan) and enzyme (green). The residues involved in these key hydrogen bonds showed significant combined chemical shift changes for their backbone and side chain H^N^ groups (Table S1). A and B show sites ABCD with hydrogen bonding patterns observed in the NAG4_1 simulation. C shows sites CDEF and D shows sites CDE^u^F^u^ with hydrogen bonding patterns observed in the NAG4_3 simulation [Color figure can be viewed at http://wileyonlinelibrary.com]

The total nonbonded interaction energy (van der Waals and electrostatic) between the individual sugars and the protein were compared in the four NAG4 simulations (Figure S4). For the NAG4_1 simulation, the highest contributions are from subsite A (−194 ± 6 kJ/mol) and subsite C (−128 ± 1 kJ/mol) with a total non‐bonded interaction energy of −499 ± 9 kJ/mol. The sugar binding to λ lysozyme in the NAG4_2 simulation was not stable as the sugar dissociated from the active site after 100 ns (Figure S5D), the apo enzyme then adopting a closed conformation (Figure S5C). Prior to the loss of the sugar the total non‐bonded interaction energy was approximately −300 kJ/mol with most of the contributions coming from subsites B and C. In the NAG4_3 simulation, the total interaction energy is −401 ± 19 kJ/mol with the largest contribution coming from sugar 3 in subsite C (−149 ± 6 kJ/mol). In this simulation after 40 ns a similar change in the orientation of the linkage between the sugar units populating the D and E subsites is observed as is seen in the simulation NAG6_1 (compare Figure 4C and E). This makes the final two sugars more flexible and the total interaction energy increases as the final sugar starts to form hydrogen bonds with Gln 134 in the upper lip region populating sites E^u^ and F^u^ (Figure 5D; Figure S5F). In simulation NAG4_4 the total interaction energy is −545 ± 8 kJ/mol with the greatest contribution coming from the sugar in subsite E′ (−219 ± 7 kJ/mol). In this case the E′F′ subsites are right under the upper lip and the D′‐E′ linkage is making a close contact with the active site. In this simulation after approximately 70 ns the total interaction energy dropped to −300 kJ/mol but it then increased again. This behavior can be explained by the rearrangement of the lower lip region as can be seen from Figure S6. After rearrangement the sugar in sites C′ and F′ make a favorable interaction resulting in an increase in the total interaction energy. Comparison of all the simulations of λ lysozyme with NAG4 suggests that NAG4 may bind in subsites ABCD, CDEF and transiently in subsites C′D′E′F′ but not in subsites BCDE. It is interesting that the highest contributions to the interaction energy consistently correspond to the A, C, and E sites to which NAG binds in the natural NAG/NAM substrate.

In both the NAG6_1 and the NAG4_3 simulations there is a similar change in orientation of the D‐E linkage between the sugar units populating the D and E subsites. There is considerable interest in the ligand‐binding modes in subsites D and E since the catalysis of the cleavage of the β(1 → 4) glycosidic bond happens between these two subsites. We identify here changes at the glycosidic linkage at these subsites which enable the adoption of other energetically favorable states during the MD simulation in Figure 4. In the second energetically favorable state, the sugar makes favorable hydrogen bonding contacts with residues in the upper lip region of the protein.

λ lysozyme showed different conformational states in the MD simulations, especially in the upper and lower lip regions. To characterize this, the distance between the upper lip (Tyr132: Cα) and lower lip (Lys58: Cα) and their distances to the catalytic residue Glu19, which is located between them, was calculated to assess the contribution of each lip region to the opening and closing mechanism of the protein (Figures S5, S6, and S8). In addition, all the conformers of λ lysozyme from the six MD simulations were clustered: clusters 1 and 2 correspond to the open and closed conformations of λ lysozyme, respectively, and clusters 3, 4, and 5 represent conformations which are in between open and closed configurations (Figure S7). In the NAG4 simulations, which were started from the open configuration, the protein was either in the open configuration or populated conformations which are in between open and closed but the structure did not completely close. For the NAG6_1 simulation, which was started from the open conformation, almost half the conformers belong to cluster 2, indicating a closed state. The other half of the frames were distributed between the rest of the clusters indicating that the protein was closing and opening during the simulation as illustrated by the distances in Figure S8. The protein remains closed in the NAG6_2 simulation, indeed closing further than the closed crystal structure (Figure S8). A superimposition of the structures from the central members of the clusters showed that during opening of the structure there are changes to the α6 helix region (Figure S9A).

### 3.4 Comparison of the NMR data, MD simulations and crystal structures

The combined ^1^H^N^ and ^15^N chemical shift changes in the NAG4 and NAG6 NMR titrations were mapped on to the λ lysozyme complex conformations populated during the different simulations (Figure S10). On the basis of the MD simulation results we make the assumption that NAG4 can occupy both the ABCD subsites and the CDEF subsites and so in the NMR experiments we see an average which corresponds to all the subsites being occupied to some extent. To simplify the comparisons, the chemical shift data from both the NMR titration experiments were compared with the NAG6 simulations. Significant chemical shift changes or resonance broadening could reflect changes in chemical environment due to the proximity of the sugar, hydrogen bonding to a sugar unit, the presence of multiple conformations, or changes to the dynamics of the protein as a result of the sugar binding. Overall, the residues whose chemical shifts are most affected by the sugar binding surround the active‐site cleft.

Table S1 identifies hydrogen bonds between λ lysozyme and NAG6 in the crystal structure and also hydrogen bonds between the enzyme and the sugar in the two simulations of the λ lysozyme‐NAG6 complex (open and closed) and compares them with the overall chemical shift changes seen in the NAG4 and NAG6 titrations. A similar pattern of hydrogen bonding is also observed in the NAG4 simulations (Figure 5). Table S1 shows that all the residues that show direct interactions with the sugar in the crystal structure or simulations either broaden beyond detection or have a significant chemical shift change during the titrations. High hydrogen bonding populations were observed in either or both the open and closed simulations between the sugars in subsites B, C, D, and λ lysozyme, particularly involving residues Leu70, Gln98, Asn122, Ile123, and Ala125 (and also the side chain of Tyr 77). These residues are also involved in hydrogen bonding interactions in the crystal structure of the λ lysozyme‐NAG6 complex. The backbone resonances of Leu70, Asn122, Ile123, and Ala125 and the side chain of Gln98, all broaden beyond detection during the NAG4 and NAG6 titrations. The resonances of the Asn122 side chain protons also shift significantly.

As discussed above, flexibility of the linkage between the sugars occupying subsites D and E was identified in some of the simulations causing the final two sugar units to adopt alternative conformations populating subsites E^u^ and F^u^. The population of these alternative subsites, in addition to the E and F subsites, is supported by the results of the NMR titrations. Some of the residues with which the sugar units in subsites E^u^ and F^u^ interact show either significant chemical shift changes or their resonances broaden and disappear from the spectra. The largest effect is seen for Gly133 which showed a combined chemical shift change of 0.150 ppm in the NAG4 titration and in the NAG6 titration its resonance broadened and disappeared. This residue also makes hydrogen bonding interactions with the last sugar (F^u^) as illustrated in Figure 5D.

When a carbonyl group forms a hydrogen bond to the sugar, the largest effect is often seen for the amide group of the following residue rather than the amide group of the same residue. This is seen, for example, for residue Gln68 where its backbone oxygen is involved in hydrogen bonding in the simulations. Its overall backbone shifts were 0.079 and 0.096 ppm while the following residue, Leu69, showed larger shifts of 0.114 and 0.161 ppm in the NAG4 and NAG6 titrations, respectively. Interestingly, in the crystal structure Gln68 hydrogen bonds to the sugar using its side chain oxygen, not its backbone carbonyl oxygen.

The region with the most significant broadening during the titrations is the upper lip region (residues 128‐141). Some of the residues in this region, Tyr132 and Phe135, interact with the ligand in both the MD simulations and the crystal structure of the complex. It was noted earlier that some of the broadened residues do not make contact with the sugar in the crystal structure; contacts between these residues and the sugar are also not observed in the MD simulations. The broadening and disappearance of resonances for these residues in the upper lip region may therefore reflect changes in the population and dynamics of the multiple conformations this region samples in solution in the presence of inhibitor.

The chemical shift changes observed for the NAG4 and NAG6 titrations are generally similar although there are some interesting differences (Figure 3). Many of the residues that make contact with sugars in sites C and D show shifts of similar magnitudes for the two sugars; examples of this include Lys60, Leu69, Ile123, and Ala125. Interestingly, the shifts observed for Tyr77, Glu101, and Arg102 are smaller in the NAG4 titration than in the NAG6 titration; these residues make contact with sugars in the A and B sites. A similar pattern is observed for residues Ser18, Gly20, Ser26, and Phe135, which make contact with sugars in the E and F sites. NAG6 will occupy sites A‐F while NAG4 occupies either ABCD or CDEF. Therefore, in the NAG4 titration, the AB and EF sites are not fully occupied in contrast to the CD sites; this correlates well with the observed shift changes.

### 3.5 Modeling of the λ lysozyme‐peptidoglycan complex

Although there have been many studies of the chemical structure of peptidoglycans, there is no consensus on the preferred 3D molecular structure adopted or its arrangement in the cell wall.^33^, ^34^, ^35^, ^36^, ^37^ While the glycan backbone of peptidoglycan is conserved in all bacteria with alternating NAM and NAG units with a β (1–4) linkage, the peptide moiety shows diversity. Most of the NAM residues in the peptidoglycan are substituted with a pentapeptide of general sequence L‐Ala^1^‐γ‐D‐Glu^2^‐L‐Lys^3^‐D‐Ala^4^‐D‐Ala^5^. However, the residue at position 3 is replaced by diaminopimelic acid in Dap‐type peptidoglycan which is mostly seen in Gram‐negative bacteria. Here, we modeled a peptidoglycan octamer with a NAG‐NAM(‐L‐Ala‐γ‐D‐Glu‐L‐Dap‐D‐Ala‐D‐Ala) disaccharide repeating unit since λ lysozyme has been shown to be more active for gram‐negative bacterial cell wall lysis.^38^ According to theoretical and experimental analysis, the glycosidic dihedral angles of the NAM‐β(1–4)‐NAG unit are similar to a NAG‐β(1–4)‐NAG unit (chitin‐like structure) whereas the conformational map is more restricted in a NAG‐β(1–4)‐NAM unit.^37^ Therefore, we used the *φ*, *ψ*:−84°, 102° glycosidic angles for the NAM‐NAG disaccharide, which have been previously determined from free‐energy calculations.^16^ Glycosidic angles for the NAG‐NAM disaccharide were taken from the NMR structure of a peptidoglycan monomer fragment^36^ (*φ*, *ψ*:−62°, 118° where *φ* is the O5‐C1‐O4‐C4 dihedral and *ψ* is the C1‐O4‐C4‐C3 dihedral). The pentapeptide conformation seen in the NMR structures of the unbound peptidoglycan monomer, dimer, and protein‐bound peptidoglycan polymers show that it is not well‐defined.^35^, ^36^, ^39^, ^40^, ^41^ We attached the pentapeptide to each NAM unit with dihedral angles of 30° for C2‐C3‐O3‐C9 and 60° for O3‐C9‐C10‐N2 as seen in the NMR structure of the peptidoglycan dimer. After building the NAG1‐NAM1(‐P1)‐NAG2‐NAM2(‐P2)‐NAG3‐NAM3(‐P3)‐NAG4‐NAM4(‐P4) unit, energy minimization was applied in the λ lysozyme active site using the open conformation of the protein. Upon minimization, the glycosidic dihedral angles of the glycan portion did not change significantly but the pentapeptide moieties of the NAM residues showed changes in conformation.

**Figure 6:**
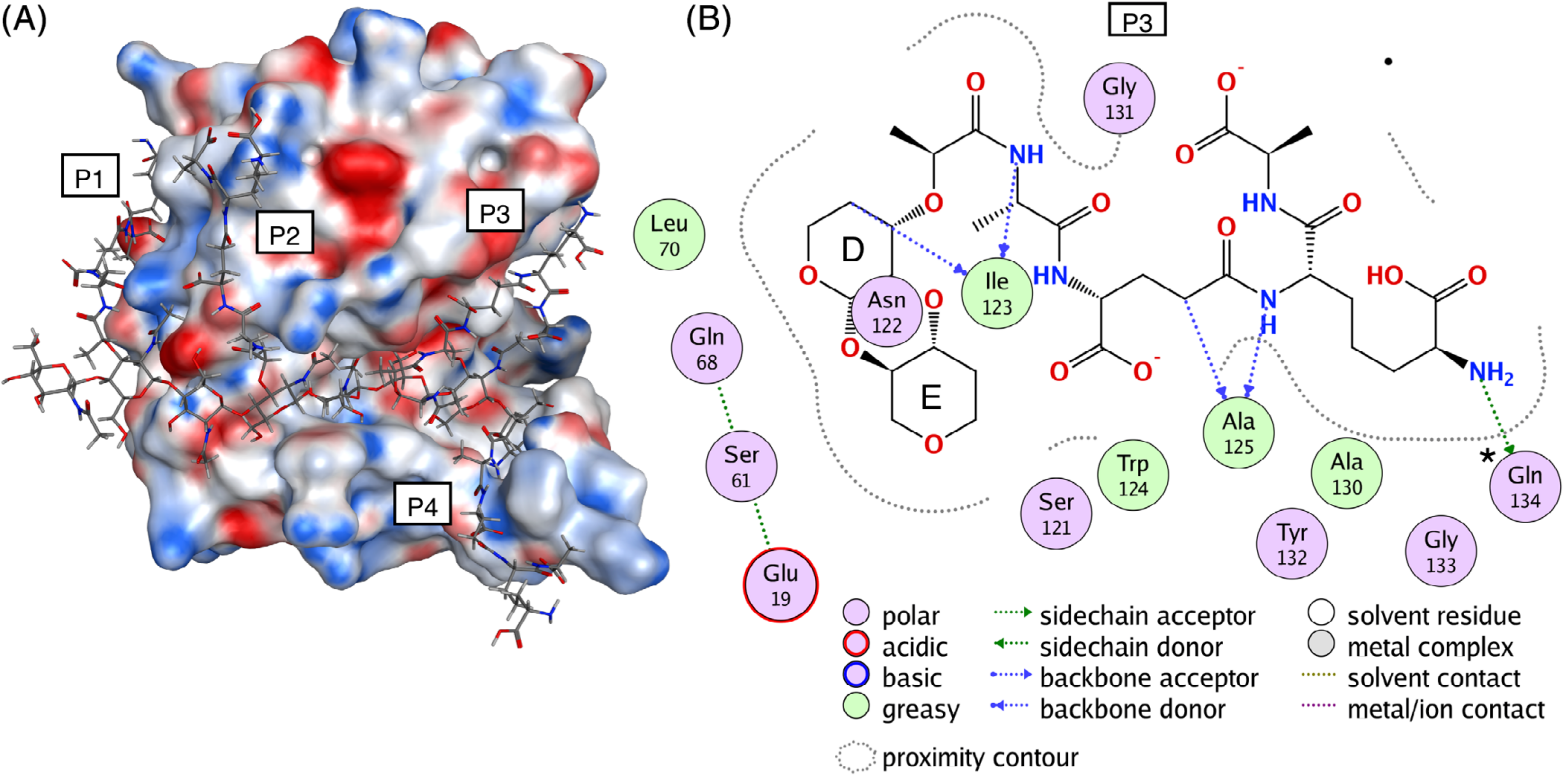
λ lysozyme‐peptidoglycan complex. A, λ lysozyme‐peptidoglycan complex model represented with the molecular surface of λ lysozyme colored according to electrostatic potential from the Poisson Boltzmann equation (peptidoglycan is NAG1‐NAM1(‐P1)‐NAG2‐NAM2(‐P2)‐NAG3‐NAM3(‐P3)‐NAG4‐NAM4(‐P4), where the peptide moieties are labeled P1, P2, P3, and P4). B, Residues of λ lysozyme which interact with the P3 pentapeptide moiety of the peptidoglycan are shown color‐coded according to the nature of the interaction (created with interaction diagram of MOE^42^). Ile123, Ala125, and Gln134 form hydrogen bonds with the P3 unit. Gln134 also forms strong hydrogen bonding interactions with the sugar in the NAG4_3 and NAG6_1 simulations when significant glycosidic angle changes are observed [Color figure can be viewed at http://wileyonlinelibrary.com]

As can be seen from the molecular surface representation (Figure 6A), after the minimization of the modeled peptidoglycan structure, the glycan portion filled the extended active‐site groove. Most of the pockets with polar sites on the protein surface identified in the MD simulations of λ lysozyme with NAG4 or NAG6, in addition to the active‐site groove, are now occupied by the peptide portion of the peptidoglycan (Figures S11 and S12). All the peptide units show favorable hydrogen bonding patterns with residues found to be important in interactions with the sugars in the simulations (Figure 6; Figures S11 and S12). Peptide moiety P3, which is in close proximity to the active site, showed strong hydrogen bonding with three residues: Ile123, Ala125 and Gln134. Interestingly, these are some of the residues which were driving the NAG6 and NAG4 sugars (without peptide chains), in the simulations NAG6_1 and NAG4_3, toward the pocket containing the E^u^ and F^u^ sugar binding sites when the structure was open. Our λ lysozyme‐peptidoglycan model suggests that in the presence of the natural substrate this E^u^F^u^ pocket will be occupied by the peptide moiety and the sugars occupy the EF or E′F′ subsites. In addition to that, our model suggests that the peptide moiety P4 binds adjacent to the λ lysozyme lower lip region. Here there was a significant loss of secondary structure in the β3‐β4 strands (residues 52‐56 and 59‐61) during the simulations. However, the model suggests that hydrogen bonding interactions between the peptide moiety P4 and Lys60 and Ser61 in λ lysozyme may stabilize the secondary structure in the lower lip in the complex with peptidoglycan. The additional interactions between these peptide moieties and λ lysozyme are likely to be responsible for the higher affinity of the enzyme for its natural substrate.

Notably, a previous structural alignment of lysozyme from T4 bacteriophage (T4L) with a dimer portion of peptidoglycan^40^ identified a similar interaction pattern between the peptidoglycan and protein close to the enzyme active site. Superimposing these structures demonstrates that the peptide moiety of peptidoglycan that makes key interactions with the T4L enzyme, occupies similar binding sites to those observed in our model of the λ lysozyme complex with the peptidoglycan octamer (Figure 6A).

## 4 CONCLUSION

NMR studies and MD simulations have given a consistent picture of the binding of NAG4 and NAG6 to λ lysozyme in solution. Residues particularly in subsites B, C, and D of λ lysozyme make persistent hydrogen bonds to the sugar units, while flexibility in the D‐E linkage enables the final two sugar units to explore a number of binding modes including the EF, E′F′, and E^u^F^u^ subsites. The D‐E linkage is in the vicinity of the catalytic residue Glu19 and this is the site where cleavage of the glycosidic bond occurs during catalysis. The combination of persistent hydrogen bonding between the sugar and protein, but with flexibility around the D‐E linkage, may be essential features for the activity of the enzyme. Our λ lysozyme‐peptidoglycan model suggests that in the presence of the natural substrate the E^u^‐F^u^ pocket will be occupied by the peptide chains while the sugars occupy the E‐F or E′‐F′ subsites. This would hold the substrate in place while allowing a conformational change at the active site. In light of this behavior it is interesting that in the crystal structure of the complex of λ lysozyme with NAG6, a single sugar molecule does not occupy all six subsites in a single molecule of λ lysozyme. In molecule A of λ lysozyme subsites A‐D are populated by four of the NAG6 units and subsites E'F’ are populated by a second NAG6 molecule. Therefore, the D‐E linkage, which is flexible in the simulations, is missing in both the molecules in the crystal structure.

Previous NMR studies and MD simulations of λ lysozyme have identified that in the apo state in solution the upper and lower lip regions of the protein are flexible. In the crystal structure of the complex of λ lysozyme with NAG6 both molecules adopt a closed conformation. However, in all the MD simulations of the λ lysozyme complexes reported here, which were started from different conformations of the enzyme and with different sugar binding modes, the upper and lower lip regions are dynamic allowing the enzyme to adopt both open and closed conformations. In addition, in the NMR titrations of λ lysozyme with NAG4 and NAG6 significant broadening of the resonances of residues within the upper lip region is observed although some of these residues are not close to the ligand binding site. This suggests that the flexibility of the lip regions seen for the apo protein in solution is maintained, to some extent, in the complex although there may be changes to the timescale or nature of the dynamics. Our results suggest that an open form is needed for the entry of the substrate and upon ligand binding the upper and lower lips retain flexibility but can close to create a cage around the catalytic Glu19 as required for the activity of the enzyme. This study demonstrates that NMR spectroscopy combined with molecular dynamics simulations can provide information that is complementary to the static crystal structure by highlighting the dynamic nature of the enzyme‐inhibitor complex.

## Supporting information


**Appendix S1**: Supplementary Material
