# Supplementary material for: An NMR and MD study of complexes of bacteriophage lambda lysozyme with tetra‐ and hexa‐N‐acetylchitohexaose
Source: Proteins. 2019 Jul 26;88(1):82–93. doi: 10.1002/prot.25770 (PMC6916166; doi:10.1002/prot.25770)
Supplement: Supplementary file 1 — Appendix S1: Supplementary Material [file PROT-88-82-s001.pdf]

Supporting Information

An NMR and MD Study of Complexes of Bacteriophage Lambda Lysozyme with Tetra- and Hexa-N-acetylchitohexaose

Aysegul Turupcu,[a] Alice M. Bowen,[b] Alexandre Di Paolo,[c,e] André Matagne,[c] Chris Oostenbrink,[a] Christina Redfield,[d] and Lorna J. Smith*[b]

[a] Institute of Molecular Modeling and Simulation, University of Natural Resources and Life Sciences Vienna, Vienna, Austria.

[b] Department of Chemistry, University of Oxford, Oxford, U.K.

[c] Laboratoire d'Enzymologie et Repliement des Protéines, Centre d'Ingénierie des Protéines, Institut de Chimie, Université de Liège, Belgium.

[d] Department of Biochemistry, University of Oxford, Oxford U.K.

[e] Current address: Kaneka Eurogentec S.A., Biologics, Liège Science Park, Rue Bois Saint jean 14, B-4102 Seraing, Belgium

Key words: lysozymes, molecular dynamics, NMR spectroscopy, oligosaccharides, ligand binding

Abbreviations: Molecular dynamics (MD), Nuclear Magnetic Resonance (NMR), hexa-N-acetylchitohexaose (NAG6), tetra-N-acetylchitohexaose (NAG4), N-acetylmuramic acid (NAM), N-acetylglucosamine (NAG), Protein Data Bank (PDB), Biological Magnetic Resonance Data Bank (BMRB), Simple Point Charge (SPC)

Table S1. Hydrogen bond occurrences between the protein and sugar in the NAG6_1 and NAG6_2 simulations and the observed overall chemical shift changes.

**Protein atom**	**X-ray**	**NAG6_1 subsite populated sugar atom % population**	**NAG6_2 subsite populated sugar atom % population**	**Δδ NAG6 ppm**	**Δδ NAG4 ppm**
19 GLU OE1/2	+	D NAG HO6 6.0	E NAG HN2 41.8	0.085<br>(20: 0.082)	0.098<br>(20: 0.056)
55 LEU O	-	F NAG HN2 8.5	F NAG HN2 33.1	0.046	0.057
56 ASN HD21 (ND2)	-	E NAG O7 17.1	-	0.027	0.045
56 ASN HD22 (ND2)	-	E NAG O7 13.1	F NAG O3 82.4	0.027	0.045
59 LEU O	-	-	E NAG HO6 89.2	0.01<br>(60: 0.066)	0.072<br>(60: 0.072)
68 GLN O	*	D NAG HO6 16.3	-	0.079<br>(69: 0.161)	0.096<br>(69: 0.114)
68 GLN HE22 (NE2)	*	E NAG O3 14.9	E NAG O3 22.9	0.079	0.096
70 LEU H (N)	+	C NAG O7 7.2	C NAG O7 81.4	d.	d.
73 TRP HE1(NE1)	-	C NAG O7 24.4	A NAG O7 8.4	0.154	0.064
73 TRP HE1(NE1)	-	C NAG O3 9.5	-	0.154	0.064
77 TYR HH(OH)	+	B NAG O6 80.1	-	0.039	0.014
77 TYR OH	*	A NAG HN2 62.2	B NAG HO6 89.2	0.039	0.014
98 GLN OE1	+	B NAG HO6 80.9	B NAG O6 83.0	0.060	0.049
98 GLN HE21 (NE2)	*	C NAG O3 23.9	C NAG O3 27.2	d.	d.
101 GLU OE2	*	A NAG HO4 35.5	A NAG HN2 45.9	0.061	0.037
101 GLU OE2	*	A NAG HO3 11.3	A NAG HO3 35.4	0.061	0.037
101 GLU OE1	*	A NAG HO4 33.7	A NAG HN2 55.5	0.061	0.037
101 GLU OE1	*	A NAG HO3 11.4	A NAG HO3 32.2	0.061	0.037
102 ARG HH12	-	A NAG O6 7.2	-	0.131	0.081
102 ARG HH12	-	B NAG O3 6.8	-	0.131	0.081
122 ASN HD22 (ND2)	+	B NAG O7 8.8	B NAG O7 97.0	d.	d.
123 ILE O	+	C NAG HN2 66.7	C NAG HN2 97.5	d.<br>(124: d.)	d.<br>(124: d.)
125 ALA H (N)	+	D NAG O6 7.5	D NAG O6 81.5	d.	d.
132 TYR HH (OH)	*	D NAG O7 7.1	-	d.	d.
132 TYR OH	*	E NAG HN2 5.3	C NAG HO6 6.7	d.	d.
135 PHE H	+	-	F NAG O6 5.4	d.	d.

'+' the hydrogen bond is observed with the same protein atom as in the 3D3D crystal structure
'*' the hydrogen bond is observed with a different atom of the same residue in the 3D3D crystal structure
'–' the hydrogen bond is not observed in the 3D3D crystal structure
'd.' the resonance broadens beyond detection in the NMR spectra. Significant chemical shift changes for a following residue are indicated in parentheses

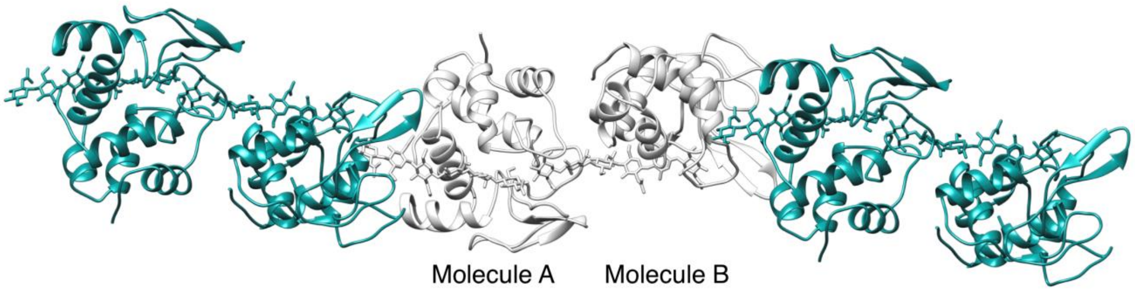

Figure S1. Unit cell of the 3D3D X-ray structure containing two molecules of the complex of λ lysozyme with NAG6 shown with the repeating units coloured in grey and cyan. Each molecule is bound to its NAG6 unit using subsites A, B, C, D and the other sugar unit using its E’ and F’ subsites.

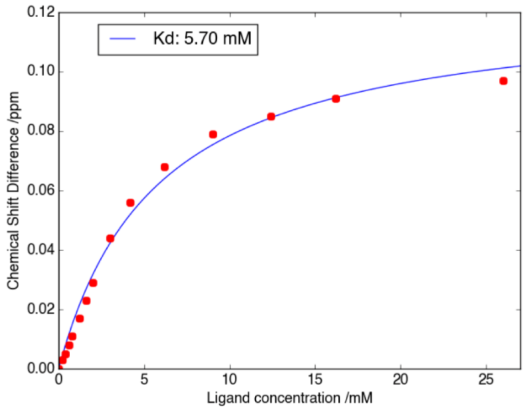

Figure S2. Plot of the titration data of λ lysozyme with NAG4. Curves were calculated using the overall ^{1}H and ^{15}N chemical shift change data from all the residues which show hydrogen bonding interaction with the sugar in the ABCDEF sites along with neighbouring residues whose chemical shifts are affected inductively (residues 19, 20, 68, 69, 73, 77, 98, 101 and 102). The K_d was determined by fitting the change in chemical shift as a function of L_T/P_T with $y = A\left((B+x) - \sqrt{(B+x)^2 - 4x}\right)$ where $A = \frac{\Delta\delta_{max}}{2}$ and $B = 1 + \frac{K_d}{P_T}$.

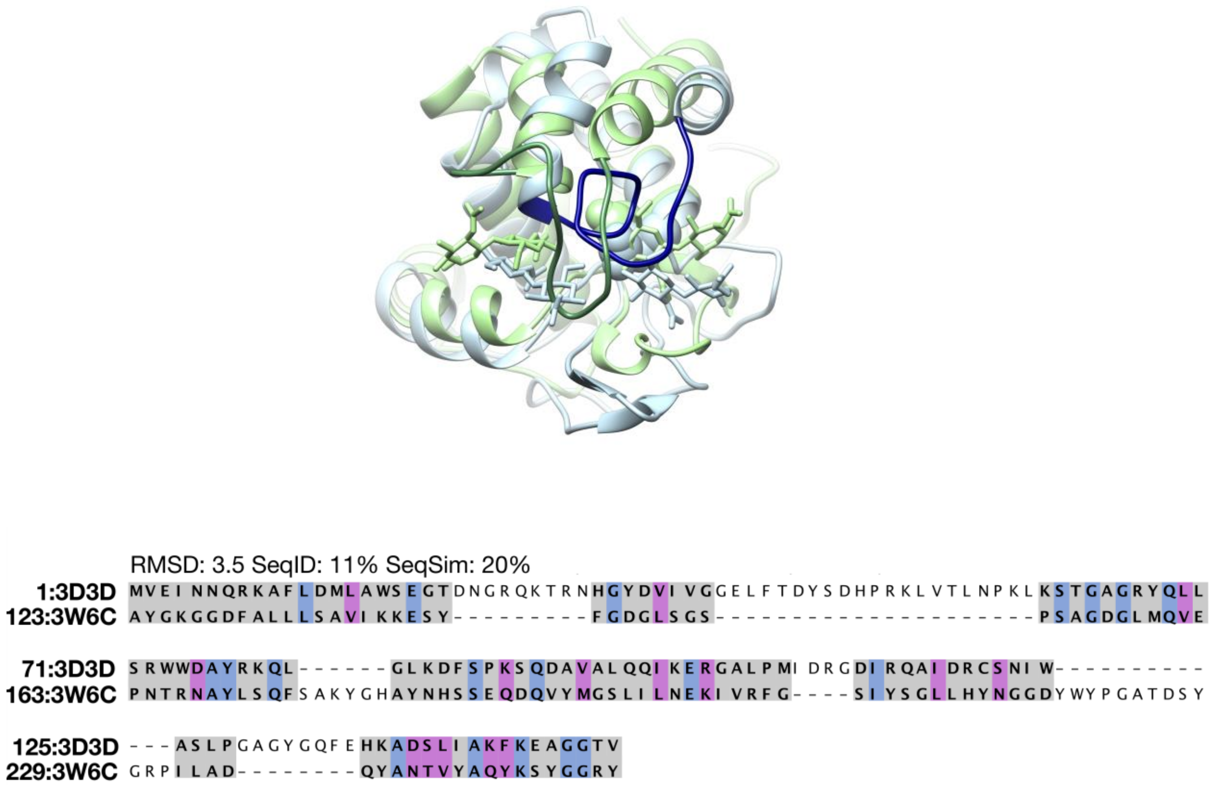

Figure S3. Structural alignment (top) of λ lysozyme (3D3D, light and dark blue) with Ra-ChiC (3W6C, light and dark green) with cleaved NAG units with an RMSD of 3.5 Å and with 20% sequence similarity. The catalytic residues Glu19 (λ lysozyme), Glu141 (Ra-ChiC) are shown as spheres which are very close to the β-hairpin motif conserved in the GH families which comprise peptidoglycan lytic transglycosylase (EC 4.2.2.n1), lysozyme type G (EC 3.2.1.17) and chitinase (EC 3.2.1.14).[2] Shared flexible loop structure enclosing the active site are emphasised with dark blue and dark green for 3D3D and 3W6C, respectively. The sequence alignment of λ lysozyme (beginning with residue 1) with Ra-ChiC (begins with residue 123) is shown in the bottom panel. Conserved residues are highlighted in blue while similar residues are highlighted in magenta.

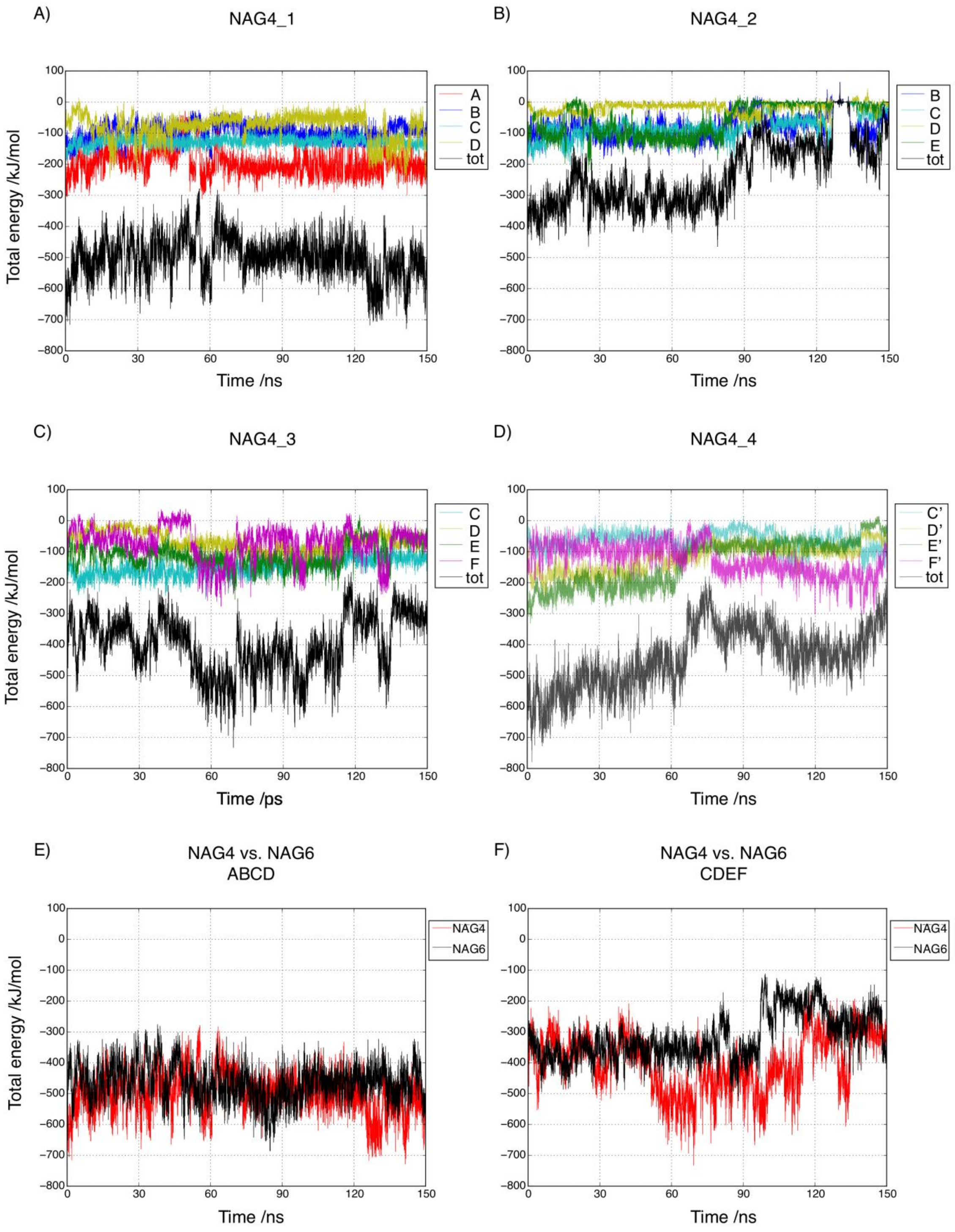

Figure S4. Time dependence of the total non-bonded interaction energy between the sugars populating individual subsites and the enzyme from the simulations of NAG4_1 (A), NAG4_2 (B), NAG4_3 (C), NAG4_4 (D). In panels A-D, total energy between the sugar and enzyme is plotted in black. In panels E and F total energy of sugars in NAG6_1 (black) and NAG4_1 (red panel E) and NAG4_3 (red panel F) is compared for sugars populating subsites ABCD and CDEF, respectively.

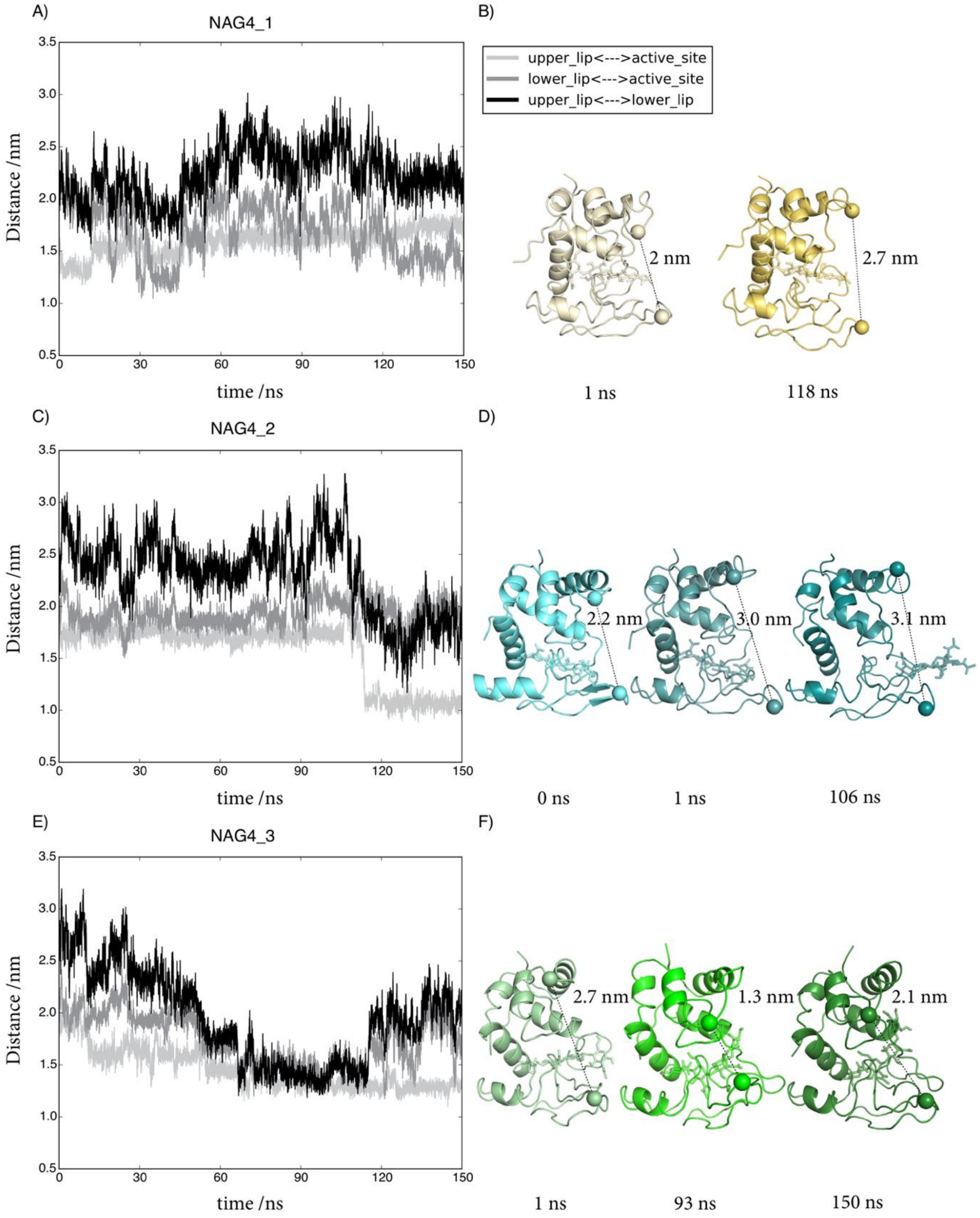

Figure S5. The upper and lower lip dynamics are illustrated by distance time series followed through the simulations of λ lysozyme with NAG4. In panels A, C and E, the distance between the Cα atom of Lys58 in the lower lip and the Cα atom of Tyr132 in the upper lip is shown in black together with the distance between the Cα atom of Lys58 in the lower lip and Glu19 in the active site cleft (light grey) and distance between the Cα atom of Tyr132 in the

upper lip and Glu19 in the active site cleft (dark grey). In panels B, D and F, conformations of λ lysozyme are shown that were extracted at selected time points during the simulations. The Cα atoms of Lys58 in the lower lip and of Tyr132 are shown as spheres with the distance between them indicated. In the NAG4_2 simulation the sugar binding was not stable and the sugar lost its interaction with the enzyme (see Figure S4) and left the catalytic site. In this NAG4_2 simulation, interaction of the sugar with sites B and E were lost initially and then there were additional conformational changes in λ lysozyme which led to a further opening of the upper lip region making the BCDE binding for NAG4 unfavourable. Further opening of the upper and lower domains was also observed in other simulations (NAG4_1, 3 and 4). However, in these simulations the interactions between the protein and sugar were strong enough to keep the sugar in the active site cleft. In the NAG4_1 simulation, site A had the biggest contribution to the total interaction energy and in the NAG4_3 and 4 simulations further arrangement of the sugar enabled it to be kept in the cleft.

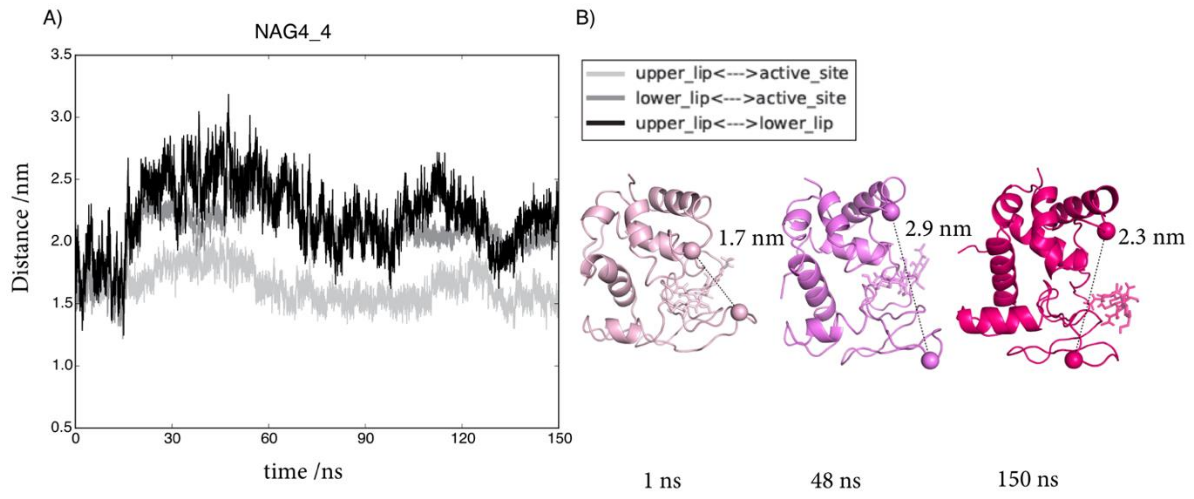

Figure S6. The upper and lower lip dynamics are illustrated by distance time series followed through the NAG4_4 simulation. In panel A the distance between the Cα atom of Lys58 in the lower lip and the Cα atom of Tyr132 in the upper lip is shown in black together with the distance between the Cα atom of Lys58 in the lower lip and Glu19 in the active site cleft (light grey) and distance between the Cα atom of Tyr132 in the upper lip and Glu19 in the active site cleft (dark grey). In panel B conformations of λ lysozyme are shown that were extracted at selected time points through the simulations. The Cα atoms of Lys58 in the lower lip and of Tyr132 are shown as spheres with the distance between them indicated.

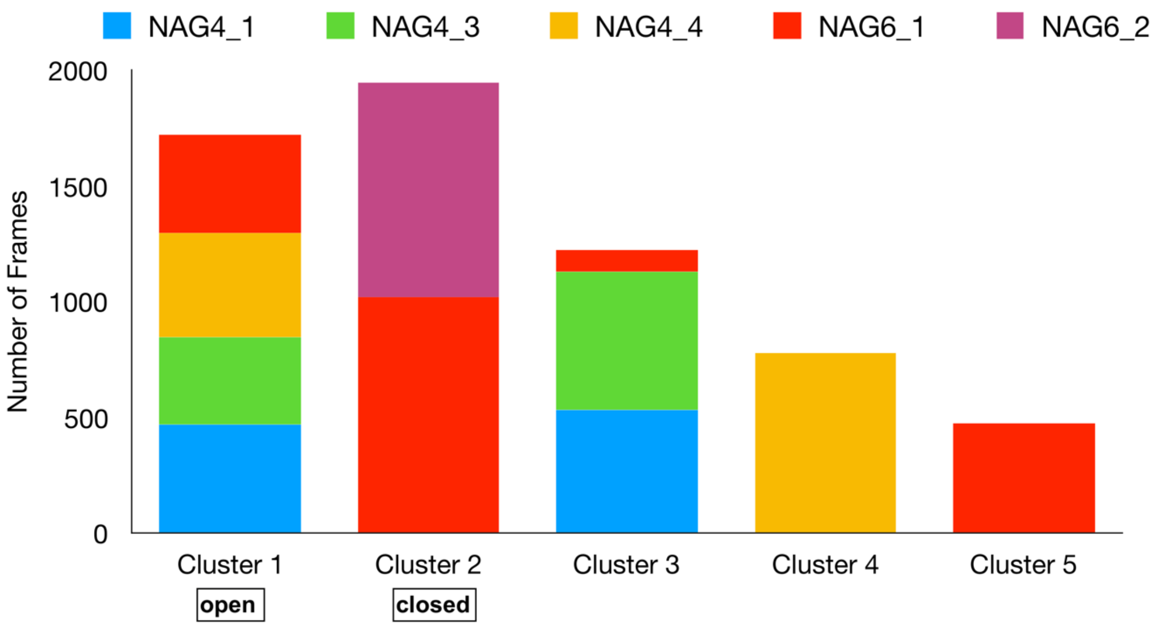

Figure S7. Clustering analysis was carried out after creating an RMSD matrix of all the frames from the NAG4 and NAG6 simulations with a 0.35 nm cutoff. Contributions of each of the simulations to the clusters are depicted with different colours. Cluster 1 represents conformations similar to the open crystal structure and cluster 2 conformations similar to the closed structure. Clusters 3, 4 and 5 represent conformations which are in between the open and closed structures. Simulation NAG4_2 was excluded from this analysis as the inhibitor molecule dissociated from the active site.

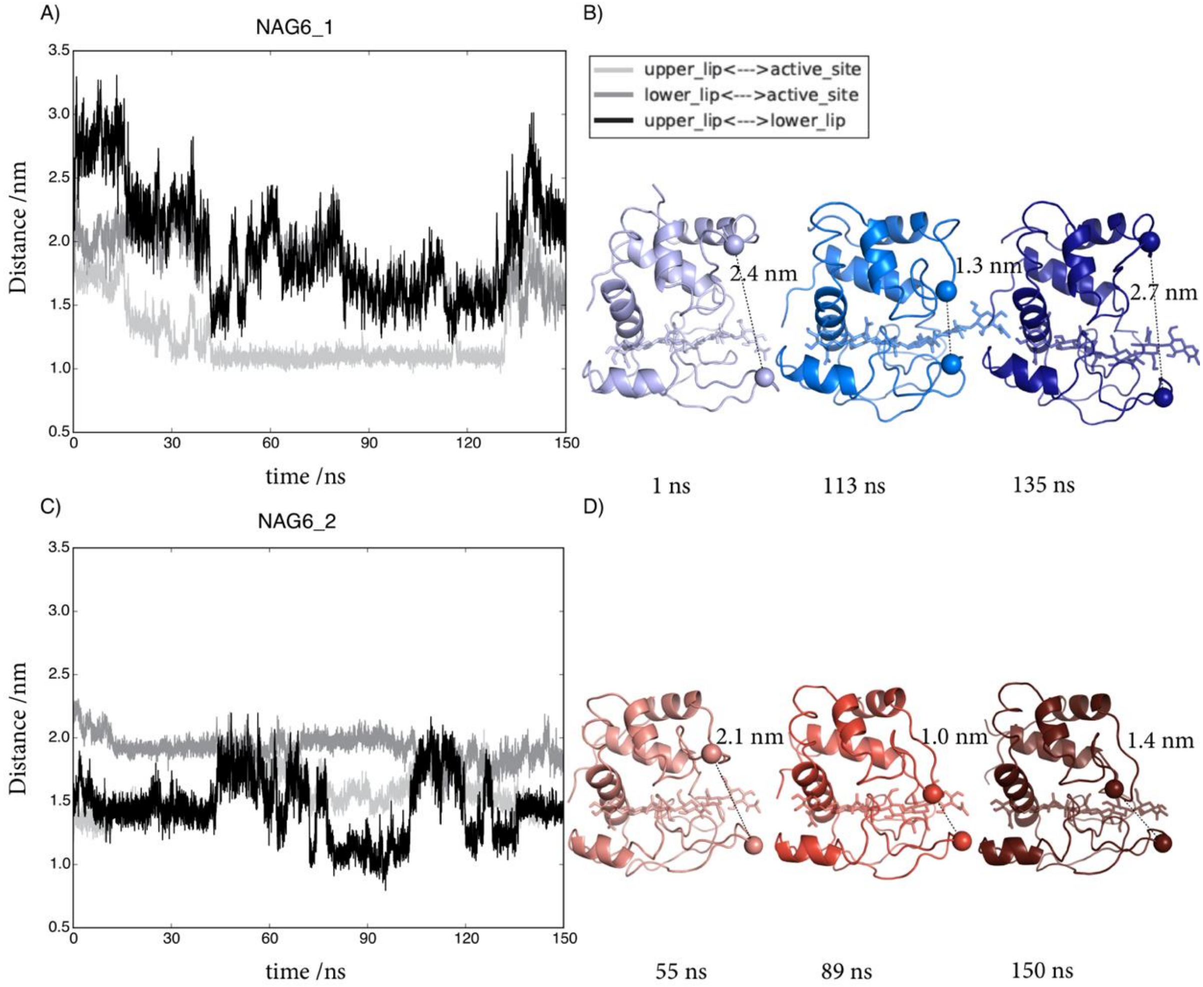

Figure S8. The upper and lower lip dynamics are illustrated by distance time series followed through the NAG6_1 and NAG6_2 simulations. In panels A and C the distance between the Cα atom of Lys58 in the lower lip and the Cα atom of Tyr132 in the upper lip is shown in black together with the distance between the Cα atom of Lys58 in the lower lip and Glu19 in the active site cleft (light grey) and distance between the Cα atom of Tyr132 in the upper lip and Glu19 in the active site cleft (dark grey). In panels B and D conformations of λ lysozyme are shown that were extracted at selected time points during the simulations. The Cα atoms of Lys58 in the lower lip and of Tyr132 are shown in spheres with the distance between them indicated. Although the opening range is smaller in the simulation where the initial structure was closed (NAG6_2), after closing of the active site it still reopens.

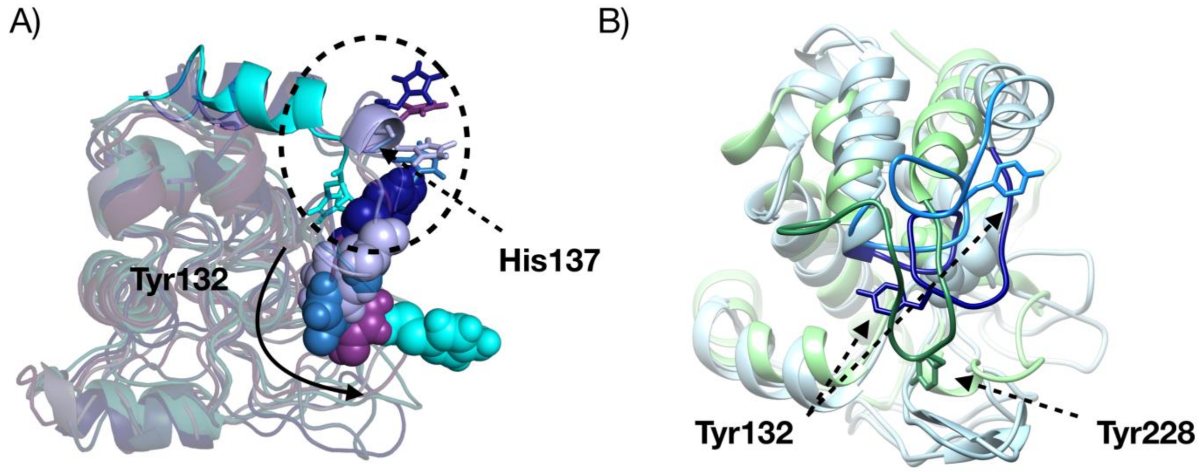

Figure S9. A) Superposition of λ lysozyme structures taken from the simulations with NAG4 and NAG6. Representative members from each of the clusters identified are aligned and coloured individually. The change in secondary structure, where the first turn of helix α6 (His 137 in sticks) unwinds, is highlighted. This results in a large overall chemical shift change for the resonances of His137 of 0.247 ppm in the λ lysozyme -NAG4 titration. The conformation of this residue is recognised to be important as its mutation causes the enzyme to be inactive.[3] Large displacements of the Tyr132 residue, which is located in the upper loop region, are also shown in spheres. B) Alignment of the Ra-ChiC structure (green) with the structure of λ lysozyme. The upper long loop in the open state and closed state is shown, emphasizing the similarity of the loop regions, which become longer upon unwinding in λ lysozyme (from light to dark blue, respectively). In the closed state of both the phenol ring of a Tyr residue (Tyr132 in λ lysozyme; Tyr228 in Ra-ChiC) is closing the active-site region like a cage.

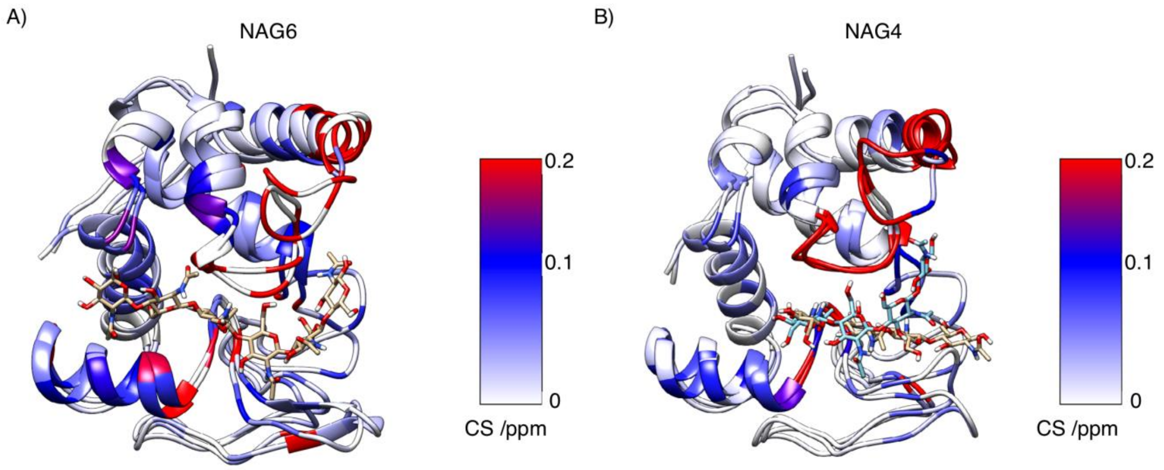

Figure S10. The combined $^{1}H^{N}$ and ^{15}N chemical shift changes observed in the λ lysozyme - NAG6 and NAG4 NMR titrations are mapped on to the structure of λ lysozyme. The colouring key is shown on the right. The residues for which the NMR resonances broadened beyond detection are set to the maximum chemical shift change and are coloured in red; residues with chemical shifts lower than the standard deviation along with Pro residues are coloured in white. A) Two different upper lip conformations taken from the NAG6_1 MD simulation were aligned to depict the residues involved in the flexibility of the lip regions. B) Different conformations of the λ lysozyme -NAG4 complex taken from the NAG4_3 MD simulation are illustrated with stick representation where the starting conformation is coloured in brown.

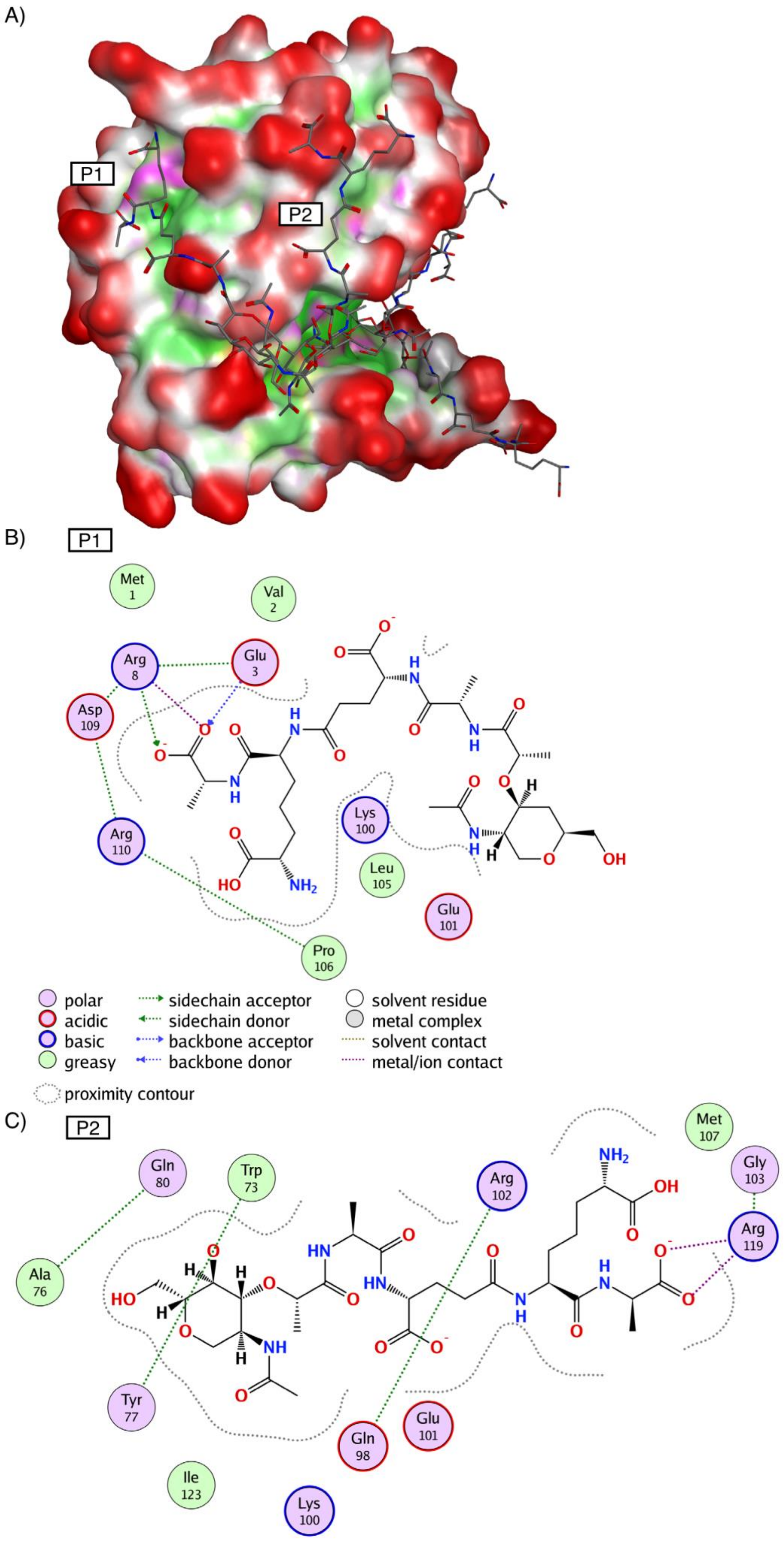
A)
P1
P2
B)
P1
Met 1
Val 2
Arg 8
Glu 3
Asp 109
Arg 110
Lys 100
Leu 105
Glu 101
Pro 106
polar
acidic
basic
greasy
sidechain acceptor
sidechain donor
backbone acceptor
backbone donor
solvent residue
metal complex
solvent contact
metal/ion contact
proximity contour
C)
P2
Gln 80
Trp 73
Arg 102
Met 107
Gly 103
Arg 119
Ala 76
Tyr 77
Gln 98
Glu 101
Ile 123
Lys 100

Figure S11. The λ lysozyme - peptidoglycan complex model concentrating on the interactions with the P1 and P2 pentapeptide part of the peptidoglycan. A) The λ lysozyme molecular surface is coloured by the property of the pockets; polar regions in magenta, hydrophobic regions in green and exposed regions are in red. Residues of λ lysozyme which are interacting with the P1 (B) and P2 (C) pentapeptide parts of the peptidoglycan are analysed with MOE.[1]

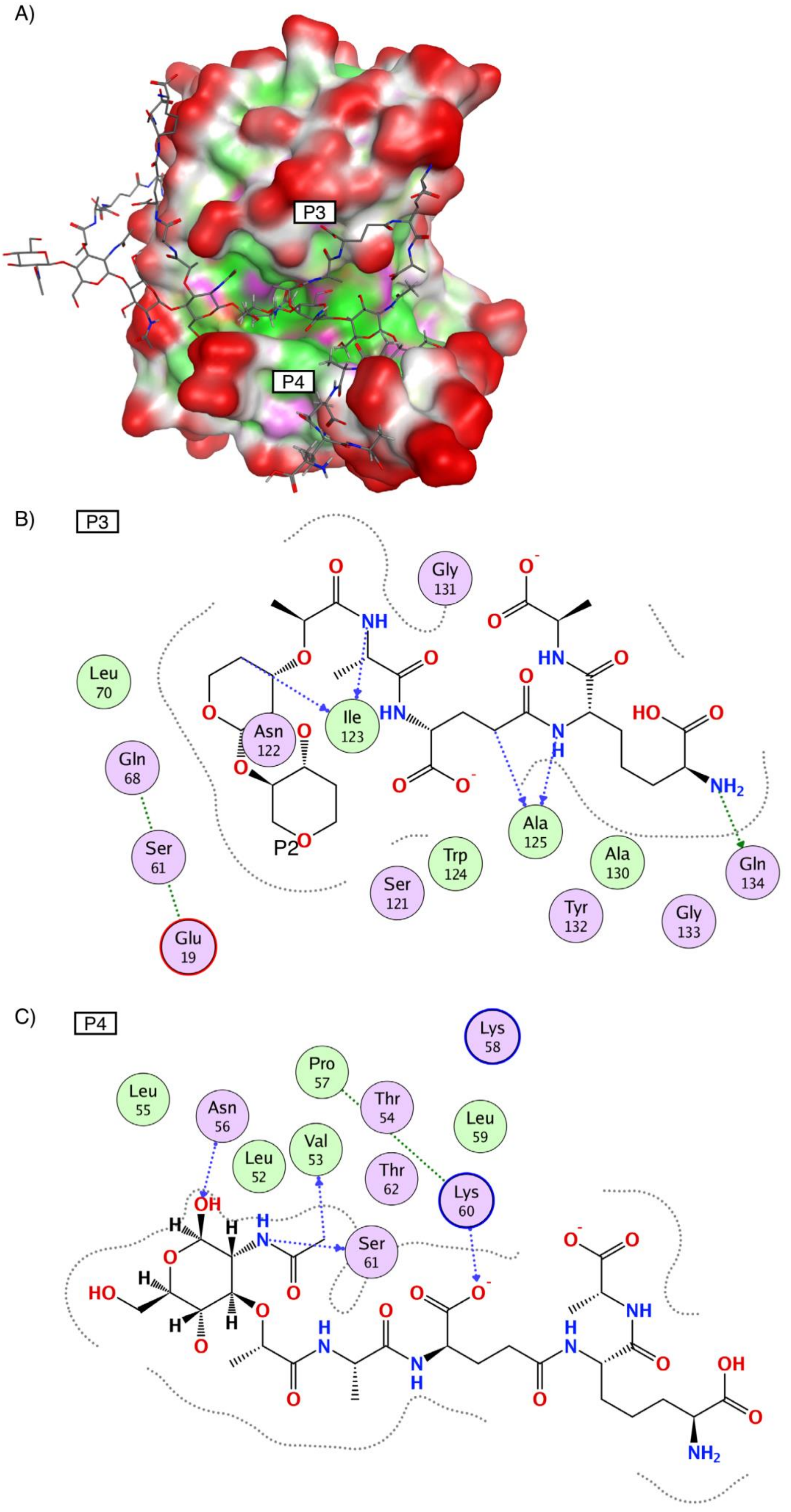
A)
P3
P4
B)
P3
Gly 131
Leu 70
Ile 123
Asn 122
Gln 68
Ser 61
Glu 19
P2
Ala 125
Trp 124
Ser 121
Ala 130
Tyr 132
Gly 133
Gln 134
HO
NH2
C)
P4
Lys 58
Pro 57
Leu 55
Asn 56
Thr 54
Leu 59
Leu 52
Val 53
Thr 62
Lys 60
Ser 61
OH
HO
NH2

Figure S12. The λ lysozyme - peptidoglycan complex model concentrating on the interactions with the P3 and P4 pentapeptide part of peptidoglycan. A) The λ lysozyme molecular surface is coloured by the property of the pockets; polar regions in magenta, hydrophobic regions in green and exposed regions are in red. Residues of λ lysozyme which are interacting with the P3 (B) and P4 (C) pentapeptide parts of the peptidoglycan are analysed with MOE.[1]

REFERENCES

[1] *Molecular Operating Environment (MOE) 2013.08*, **2018**, Chemical Computing Group ULC, Montreal, QC, Canada.

[2] N. T. Blackburn, A. J. Clarke, *J. Mol. Evol.* **2001**, *52*, 78–84.

[3] C. Evrard, J. Fastrez, P. Soumillion, *FEBS Lett.* **1999**, *460*, 442–446.
